# The DDR-related gene signature with cell cycle checkpoint function predicts prognosis, immune activity, and chemoradiotherapy response in lung adenocarcinoma

**DOI:** 10.1186/s12931-022-02110-w

**Published:** 2022-07-15

**Authors:** Quan Li, Pan Zhang, Huixiao Hu, Hang Huang, Dong Pan, Guangyun Mao, Burong Hu

**Affiliations:** 1 Department of Radiation Medicine, School of Public Health and Management, Wenzhou Medical University, Wenzhou, 325035 China; 2 Zhejiang Provincial Key Laboratory of Watershed Sciences and Health, Wenzhou Medical University, Wenzhou, 325035 China; 3 South Zhejiang Institute of Radiation Medicine and Nuclear Technology, Wenzhou Medical University, Wenzhou, 325035 China; 4 Department of Dermatology, Duke University Medical Center, Durham, NC 27710 USA; 5 Department of Preventive Medicine, School of Public Health and Management, Wenzhou Medical University, Wenzhou, 325035 China

**Keywords:** Lung adenocarcinoma, Cancer prognosis, DNA damage repair, Cell cycle checkpoint, Gene signature

## Abstract

**Background:**

As a DNA surveillance mechanism, cell cycle checkpoint has recently been discovered to be closely associated with lung adenocarcinoma (LUAD) prognosis. It is also an essential link in the process of DNA damage repair (DDR) that confers resistance to radiotherapy. Whether genes that have both functions play a more crucial role in LUAD prognosis remains unclear.

**Methods:**

In this study, DDR-related genes with cell cycle checkpoint function (DCGs) were selected to investigate their effects on the prognosis of LUAD. The TCGA-LUAD cohort and two GEO external validation cohorts (GSE31210 and GSE42171) were performed to construct a prognosis model based on the least absolute shrinkage and selection operator (LASSO) regression. Patients were divided into high-risk and low-risk groups based on the model. Subsequently, the multivariate COX regression was used to construct a prognostic nomogram. The ssGSEA, CIBERSORT algorithm, TIMER tool, CMap database, and IC50 of chemotherapeutic agents were used to analyze immune activity and responsiveness to chemoradiotherapy.

**Results:**

4 DCGs were selected as prognostic signatures, and patients in the high-risk group had a lower overall survival (OS). The lower infiltration levels of immune cells and the higher expression levels of immune checkpoints appeared in the high-risk group. The damage repair pathways were upregulated, and chemotherapeutic agent sensitivity was poor in the high-risk group.

**Conclusions:**

The 4-DCGs signature prognosis model we constructed could predict the survival rate, immune activity, and chemoradiotherapy responsiveness of LUAD patients.

**Supplementary Information:**

The online version contains supplementary material available at 10.1186/s12931-022-02110-w.

## Background

Cancer is the primary threat to human public health, with an estimated 23.6 million new cancer cases and 10 million cancer deaths by 2019 worldwide [1]. Lung adenocarcinoma (LUAD), the primary pathological type of lung cancer, remains the predominant cause of cancer deaths in most countries [2, 3]. Despite significant breakthroughs achieved in the treatment of LUAD, radiation and drug resistance after treatments are still the main challenges to the survival of patients [4]. Emerging studies suggest that autophagy-related signature [5], immune-related signature [6], and methylation-related markers [7] can be used as prognostic markers to predict the prognosis of LUAD patients, while the heterogeneity of the tumor makes it difficult to evaluate the prognosis of each patient precisely [8]. Therefore, discovering and identifying a distinctive prognostic signature for LUAD to accurately assess patient outcomes and facilitate individual tumor treatment remains critical.

DNA damage can be recognized and repaired by the cell's internal DNA damage repair (DDR) mechanism. Incorrect repair is one of the leading causes of cancer’s occurrence and development [9]. Studies have shown that DDR acts as a barrier to tumorigenesis in the early stages of LUAD. Yet, it can promote malignant growth in tumor cells with defective genomic maintenance mechanisms [10]. In addition, DDR is associated with radiation resistance in LUAD cells. The serine proteinase inhibitor clade E member 2 (SERPINE2), a DDR-related gene, regulates radiation sensitivity. Its high expression positively correlates with poor prognosis in patients with LUAD [11]. ERK5 increases the radiation resistance of LUAD cells by enhancing the DNA damage response, leading to a poor prognosis for patients [12]. Ubiquitin-specific protease 14 (USP14) is a modulator of the double-strand break (DSB) repair pathway that increases radiation resistance in LUAD cells, leading to poor treatment in patients [13]. It is no doubt that DDR-related genes are closely related to LUAD prognosis.

Cell cycle checkpoints, acting as DNA surveillance mechanisms, can prevent the accumulation and propagation of genetic errors during cell division [14]. Activating different cell cycle checkpoints is also considered an essential process in DDR, allowing cells time to repair their damaged DNA before moving to the next cell cycle stage [15]. When DNA double-strand breaks (DSBs), the primary type of DNA damage, the MRE11/NBS1/RAD5 complex activates the ataxia telangiectasia mutated (ATM) /checkpoint kinase 2 (CHK2) pathway to promote the S-phase cell cycle arrest and the p53-associated G1/S-phase checkpoint [16, 17]. Furthermore, the cell cycle checkpoint is closely related to LUAD prognosis. Some studies suggested that genetic variations in the CHEK2 gene may play a key role in predicting the toxicity and prognosis of NSCLC [18]. Higher checkpoint gene PRKCSH expression, which suppresses the activation of the STAT6/p53 pathway, was correlated with a poorer prognosis and more significant infiltration of most immune cell types in patients with lung cancer [19].

As mentioned above, both DDR and cell cycle checkpoint are closely related to the prognosis of LUAD, while the cell cycle checkpoint also has a vital role in DDR. In addition, radiotherapy and chemotherapy are two of the three primary means of cancer treatments, which rely on causing DNA breaks to kill tumor cells. Therefore, the expression of genes related to DDR and cell cycle regulation also directly affect chemoradiotherapy results. A reasonable hypothesis could be generated that genes with both cell cycle checkpoint and DDR functions may have more critical effects on LUAD prognosis.

Consequently, the DDR-related genes with cell cycle checkpoint function, here called DCGs, were selected to construct a prognosis model for LUAD by a systematical method and its effect on immune activity and response to chemoradiotherapy were further explored to reveal the causes behind the poor prognosis.

## Materials and methods

### Data collection

The transcriptome profiling data and clinical information of 322 lung adenocarcinoma (tumor purity > 60) and 59 normal samples were downloaded from the TCGA database by using the R package “TCGAbiolinks”. The two independent validation cohorts, including GSE31210 (224 lung adenocarcinoma samples) and GSE42171 (181 lung adenocarcinoma samples), were downloaded from the GEO database by using the R package "GEOquery". The raw RNA-Seq transcriptome count data were normalized by the R package "EDASeq" and were log2 (data + 1) transformed for the following analysis. 296 DNA damage repair related-genes were obtained from GeneCards (https://www.genecards.org/), listed in Table S1 in Additional file 1.

### Functional enrichment analyses

To search the DDR-related genes with cell cycle checkpoint function (DCGs), the 296 DDR genes were annotated by Ontology (GO) and Kyoto Encyclopedia of Genes and Genomes (KEGG) using R packages “clusterProfiler” and “org.Hs.eg.db”.

### Identification of differentially expressed DCGs

The expression of DCGs in the TCGA cohort was analyzed to identify differentially expressed genes (DEGs) between LUAD and normal samples. The DEGs with a *P* < 0.05 and |logFC|> = 1 were determined by the R package “limma”. The heatmap and volcanic plot of DEGs were depicted by R package “pheatmap” and "EnhancedVolcano”. The Protein–protein interaction (PPI) network of DEGs was constructed by the STRING database (https://cn.string-db.org/), and the hub genes were selected by the cytohubba plugin (Degree method) in Cytoscape software.

### DNA methylation analysis

MethSurv (https://biit.cs.ut.ee/methsurv/) is a bioinformatics tool for survival analysis based on CpG methylation patterns, with methylation data of multiple human cancers [20, 21]. The CpG methylation status of DCGs and the associations between DCGs methylation, gene expression, and prognosis were revealed by using this tool in our study.

### Unsupervised consensus clustering analysis

Consensus Clustering (unsupervised clustering) is a common cancer subtype classification method that can distinguish samples into several subtypes according to different omics data sets to discover new disease subtypes or compare different subtypes [22]. Here, a Consensus Clustering analysis was performed to identify the heterogeneity of LUAD based on the expression levels of DCGs. The LUAD subtype was classified by the “K-mean” method on the R package “ConsensusClusterPlus”.

### Establishment and validation of the DCGs prognostic model

A total of 298 TCGA-LUAD samples (excluding patients with a survival time of fewer than 30 days) were randomized into a training set (n = 198) and a test set (n = 100) in a 2:1 ratio by using the R package “caret” (set seed 123,456, y = overall_survival).

The process of the prognostic model establishment was performed in the training set. The test set, external validation cohort GSE31210 and GSE42171, were used to validate the predictive capability of the prognostic model. The receiver operator characteristic (ROC) curves were constructed to assess the capacity of the prognosis model.

Univariate Cox regression analysis was first used to screen for survival-associated DCGs using the R package “survminer” and “survival”. Subsequently, the least absolute shrink-age and selection operator (LASSO) regression model was used to prevent overfitting, selecting the best candidate genes into the prognosis model by using the R package “glmnet”. A risk score was calculated by Lasso regression coefficients. $\mathrm{Risk score}=\sum_{\mathrm{i}}^{\mathrm{k}}\mathrm{Xi}\times \mathrm{Yi}$ (X: coefficients, Y: gene expression level). LUAD patients were divided into high-risk and low-risk groups based on the optimal cutoff value of the risk score using the X-tile software, and the test set and validation cohorts were applied to the same cutoff value.

Kaplan–Meier (K–M) survival curve analysis was conducted to compare the overall survival (OS) between the two groups by using the R package “survival”. Principal component analysis (PCA) based on the DCGs was conducted by the “prcomp” function in the R package “stats”. The receiver operator characteristic (ROC) curves for 1-, 3- and 5-years survival were constructed using the R package “timeROC” to assess the predictive accuracy of the DCGs prognosis model.

### Establishment of prognosis nomogram combined with clinical features

The Univariate COX regression model was conducted to analyze the risk score and clinical characteristics of TCGA-LUAD patients. Variables with *P* < 0.05 were subsequently included in the multivariate Cox regression analysis to screen independent prognosis factors in LUAD. Finally, a nomogram was established based on multivariate Cox regression analysis results for guiding the clinical decision by using the R package “rms”. The calibration curves for 1, 3, and 5 years were used to assess the predictive ability of the nomogram.

### Assessment of immune microenvironment between two risk groups

The compositions of the 22 kinds of tumor-infiltrating immune cells between two risk groups were calculated by the CIBERSORT algorithm. The Spearman correlation analysis was performed between 27 types of immune checkpoint expression and risk score by R package “ggstatsplot”. The details of 27 immune checkpoints were listed in Table S2 in Additional file 1. TIMER (https://cistrome.shinyapps.io/timer/) is an online database for comprehensively analyzing tumor-infiltrating immune cells [23, 24]. The relationship of DCGs with immune cells was further validated in the TIMER database.

### Assessment of chemoradiotherapy response between two risk groups

The scores of DNA damage repair, X-ray, and UV response were calculated by single-sample gene set enrichment analysis (ssGSEA) on the R package “gsva”. The gene sets of DNA damage repair, X-ray, and UV response were downloaded from Gene Set Enrichment Analysis (http://www.gsea-msigdb.org/gsea/index.jsp), as shown in Table S3 in Additional file 1.

The pRRophetic (https://github.com/paulgeeleher/pRRophetic) R package was used to predict the half-maximal inhibitory concentration (IC50) of chemotherapeutic agents for two risk groups. The R package was based on pre-treatment gene expression and drug sensitivity data of cancer cell lines to predict the chemotherapeutic response [25]. The parameters of tissueType were set to the “lung”. Since Connectivity Map (CMap) (https://clue.io/) establishes links between drugs, diseases, and genes by comparing gene expression profiles [26, 27]. We used the CAMP database to identify potential small molecule drugs for high-risk groups.

### Statistics

**Fig. 1:**
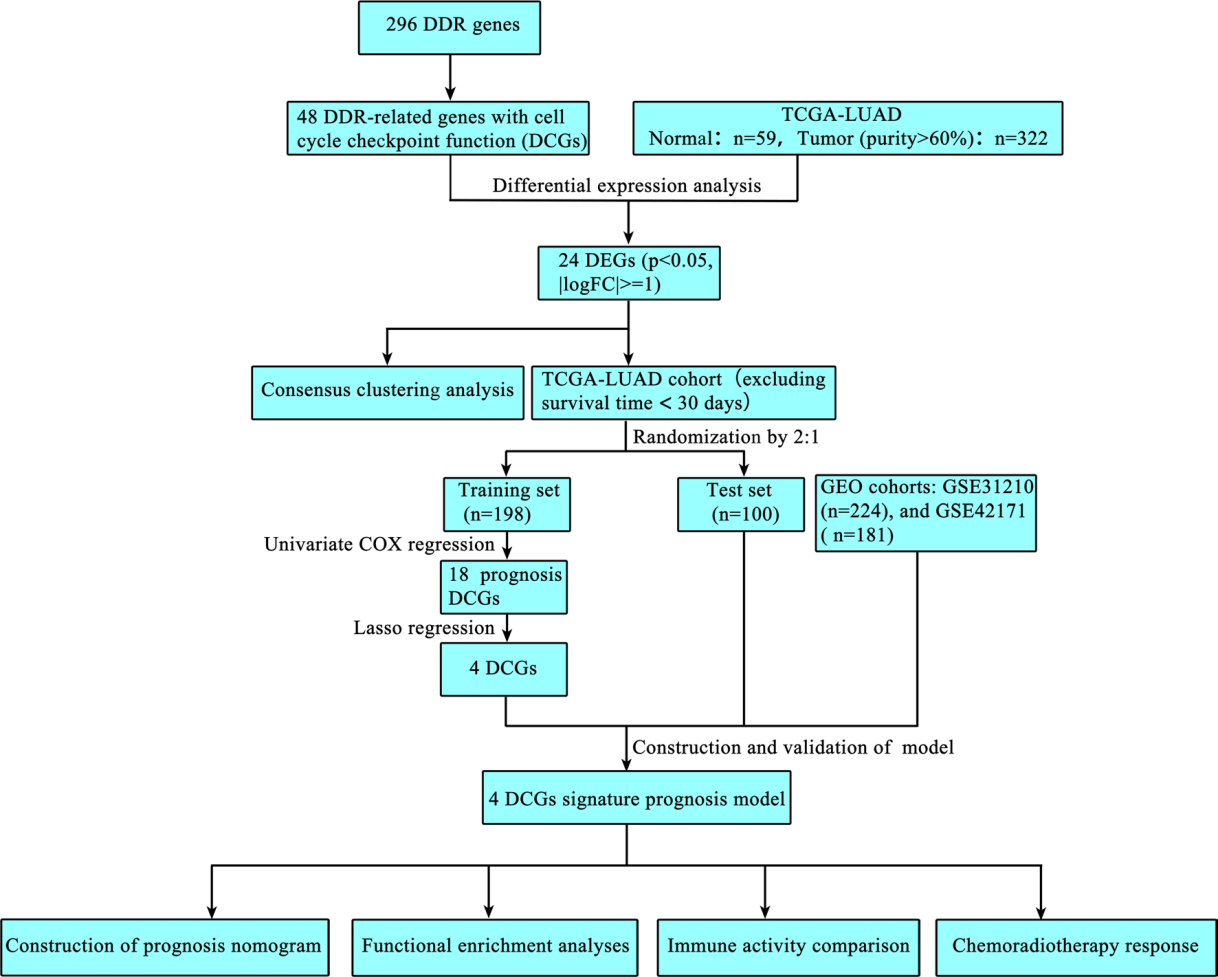
The workflow chart of this study

The t-test or the Wilcoxon test (not met parameter test requirement) were used to compare the mean between the two groups. The correlation analysis was performed using the Pearson correlation (based on bivariate normality) or Spearman correlation (not met parameter test requirement). The chi-square test was used for the comparison of the categorical variables. The survival curves of the two groups were plotted with the Kaplan–Meier method and tested by the Log-rank test. Independent prognosis factors were determined using Cox proportional hazards regression analysis. The LASSO regression was used to screen for key factors to construct the models. All statistical analyses were performed using the R software (version 4.1.2). *P*-value < 0.05 was considered a statistically significant. The workflow chart for this study is shown in Fig. 1.

## Results

### Identification of DDR-related genes with cell cycle checkpoint function (DCGs)

**Fig. 2:**
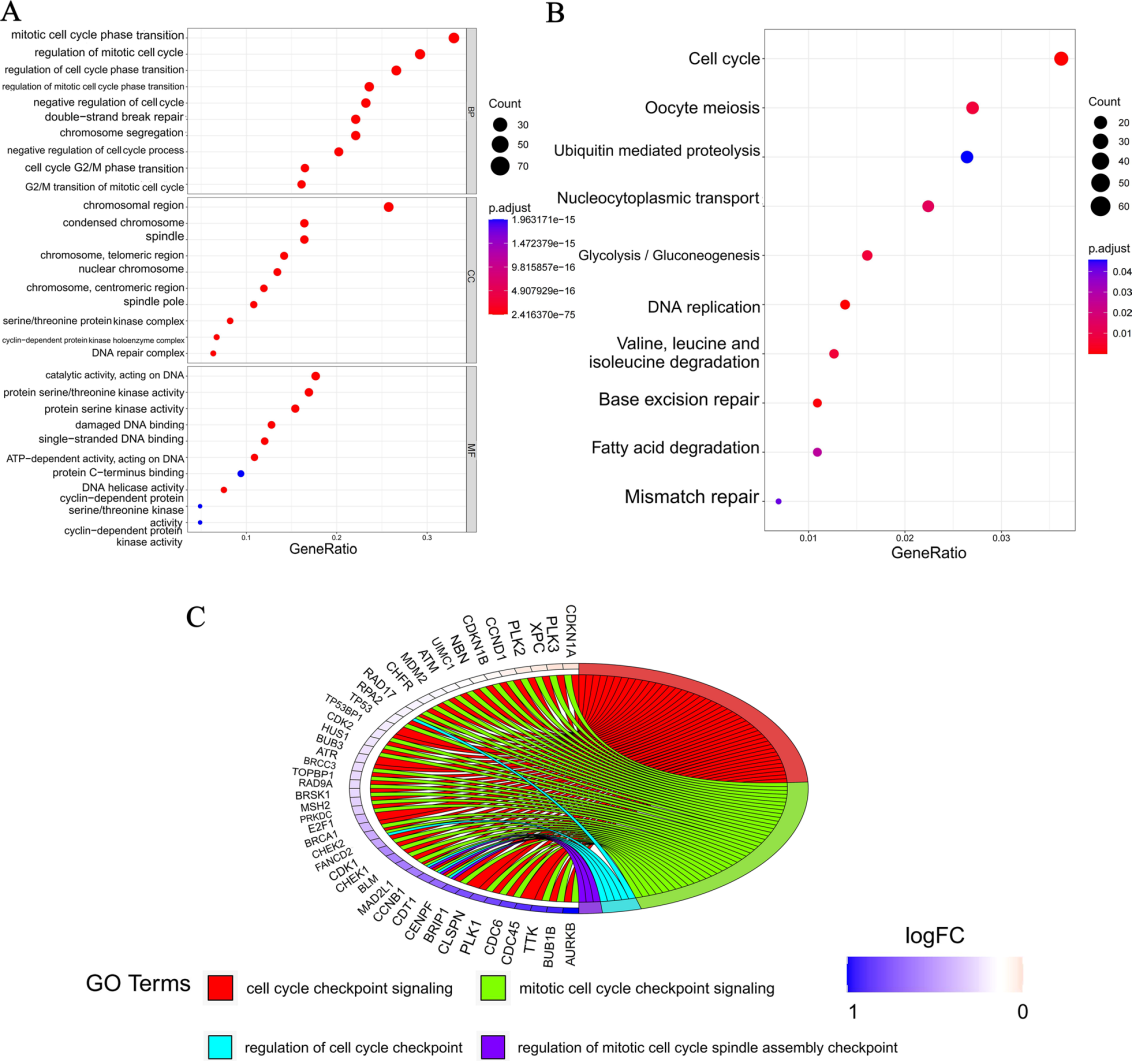
Identification of DDR-related genes with cell cycle checkpoint function (DCGs). **A** GO enrichment analysis of 296 DDR genes, BP: Biological process; MF: Molecular function; CC: Cellular components. **B** KEGG enrichment analysis of 296 DDR genes. **C** The Biological processes of cell cycle checkpoint involved by DDR genes

To explore genes with both DDR and cell cycle checkpoint functions, we performed GO and KEGG functional enrichment analyses on 296 DDR genes. The results showed that, besides being involved in DDR, these genes were also significantly enriched in the cell cycle related-regulation, such as mitotic cell cycle phase transition, regulation of mitotic cell cycle, and regulation of cell cycle phase transition, etc. (Fig. 2A, B). Further analysis of the cell cycle-related functions revealed that 44 DDR genes were significantly enriched in the cell cycle checkpoint-related biological processes (Fig. 2C). Therefore, we defined these 44 genes as DDR-related genes with cell cycle checkpoint function (DCGs).

### Identification of differentially expressed DCGs between LUAD and normal samples

**Fig. 3:**
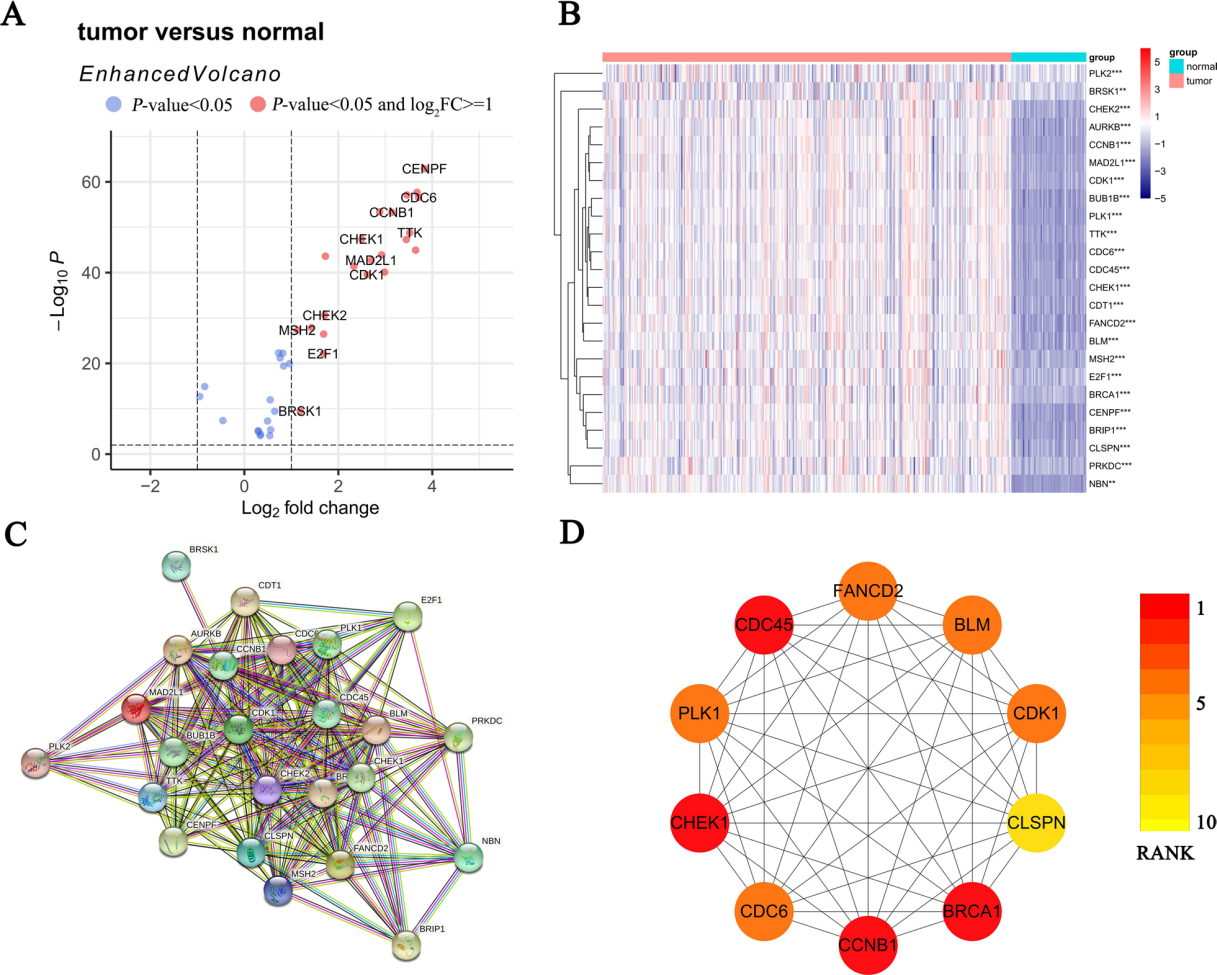
Identification of differentially expressed DCGs in TCGA-LUAD and normal samples. **A** Differential expression volcano plot based on DCGs (red: *P* < 0.05 and |logFC|> = 1, blue: only *P* < 0.05). **B** The heatmap of 24 differentially expressed DCGs in LUAD and normal samples (blue: low expression level, orange: high expression level, **P < 0.01, ***P < 0.001). **C** Protein–protein interaction (PPI) network of 22 DCGs. **D** The hub genes calculated by the PPI network

The expression levels of 44 DCGs were analyzed between TCGA-LUAD and normal samples. A total of 24 DEGs were identified (*P* < 0.05, |logFC|> = 1), and all of them (BRSK1, CDC6, FANCD2, AURKB, BUB1B, MAD2L1, PLK1, CHEK1, E2F1, MSH2, BLM, CHEK2, CDK1, BRCA1, CDT1, TTK, CCNB1, CENPF, PRKDC, BRIP1, CDC45, CLSPN, NBN, and PLK2) were upregulated in LUAD (Fig. 3A, B). Furthermore, a protein–protein interaction (PPI) for 24 DEGs was constructed (Fig. 3C), with 0.4 (medium confidence) as an interaction score standard. BRCA1, CDC45, CHEK1, CCNB1, PLK1, BLM, CDC6, CDK1, FANCD2, and CLSPN were considered hub genes based on PPI results (Fig. 3D).

### DNA methylation analysis of DCGs

**Fig. 4:**
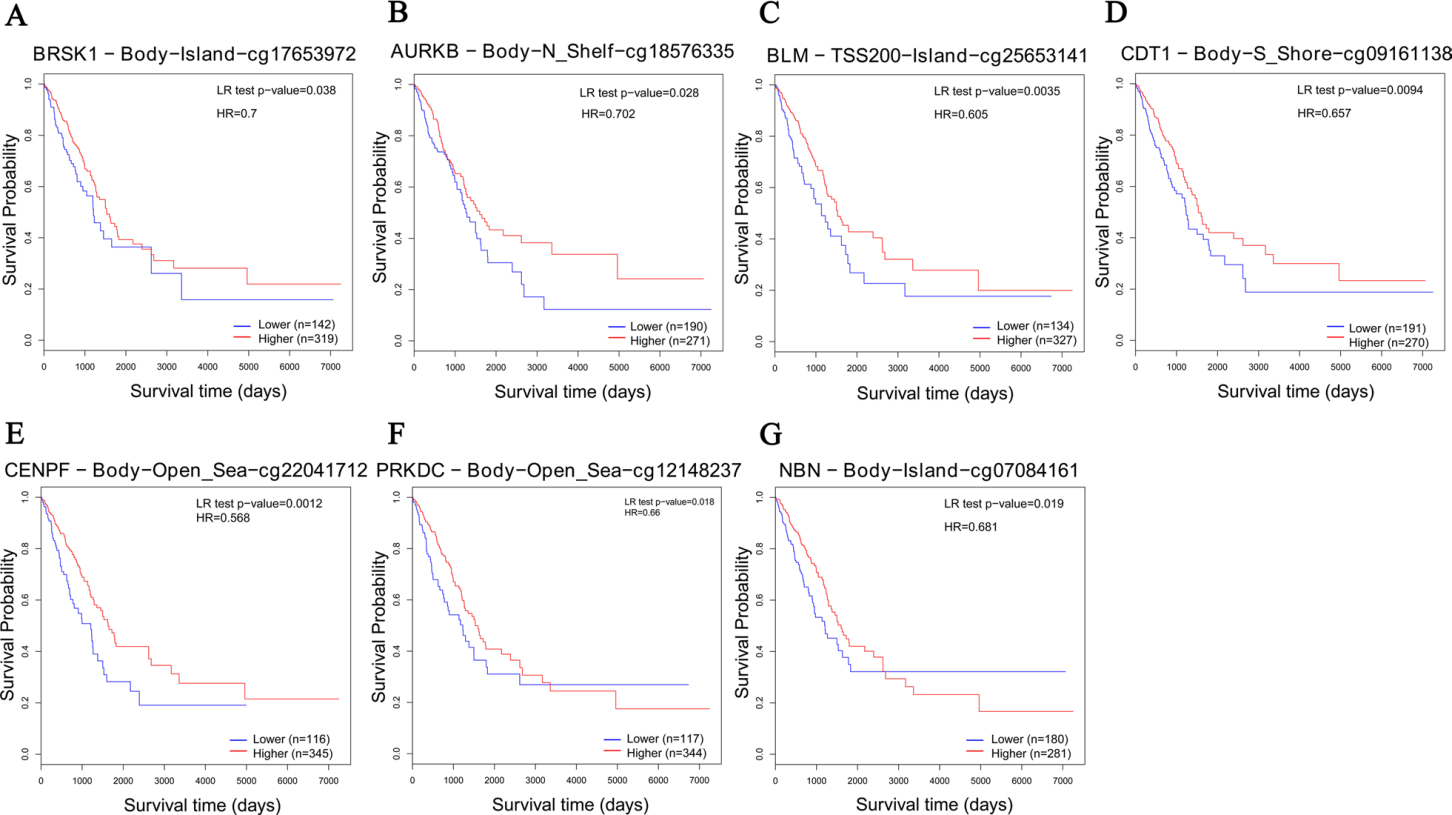
DNA methylation survival analysis of DCGs based on the MethSurv database. The Kaplan–Meier (K–M) survival curve of LUAD patients with different methylation levels in **A** cg17653972 from BRSK1, **B** cg18576335 from AURKB, **C** cg25653141 from BLM, **D** cg09161138 from CDT1, **E** cg22041712 from CENPF, **F** cg12148237 from PRKDC, and **G** cg07084161 from NBN

DNA methylation has an irreplaceable role in the gene transcriptional regulatory, and generally the methylation of the gene CpG island can exert a transcriptional silencing [28]. Therefore, to confirm the expression of DCGs in LUAD, we analyzed the methylation status of their CpG island by using the MethSurv database. The results indicated low methylation levels in most DCGs (Fig. S1 in Additional file 2). Furthermore, the low methylation levels in cg17653972 from BRSK1, cg18576335 from AURKB, cg25653141 from BLM, cg09161138 from CDT1, cg22041712 from CENPF, cg12148237 from PRKDC, and cg07084161 from NBN correlated with a poor prognosis in LUAD patients (P < 0.05, HR < 1, Fig. 4A–G).

### LUAD classification based on the DCGs

**Fig. 5:**
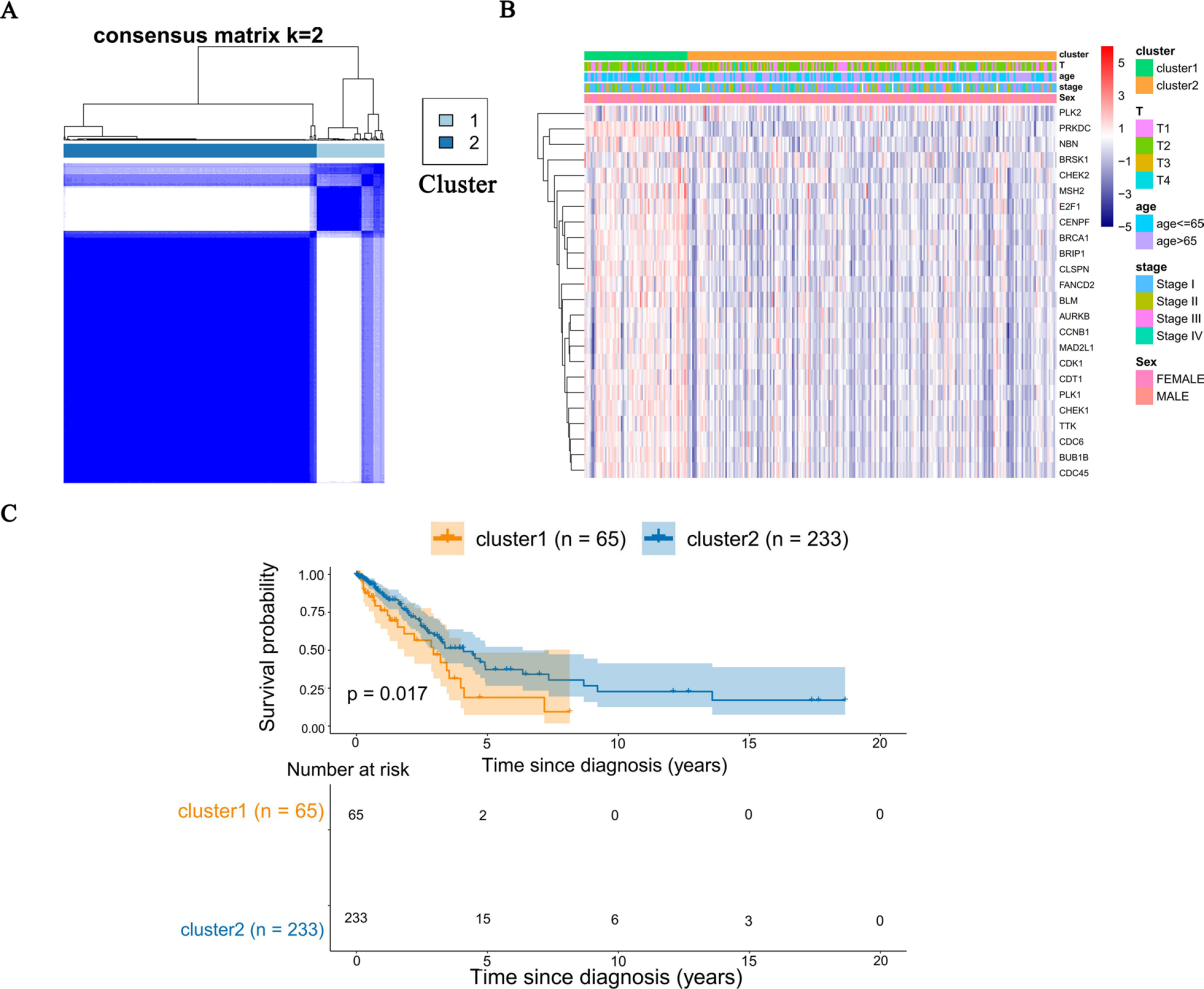
LUAD classification based on the DCGs. **A** Consensus clustering matrix for k = 2. **B** Heatmap for the expression of DCGs based on the two clusters and clinical features. **C** Kaplan–Meier OS curve for two clusters in LUAD

To investigate the heterogeneity of LUAD based on DCGs, we performed a consensus clustering analysis in TCGA-LUAD patients. K = 2 (from 2 to 5) was determined as the best cluster number, with the highest intragroup correlations (Fig. 5A). TCGA-LUAD patients were divided into two clusters according to the clustering results. The expression profile of DCGs in the two clusters combined with clinical features was shown in Fig. 5B. The heatmap displayed the difference in DCGs expression in two clusters while there was no difference in clinical features. Subsequently, survival analysis in two clusters was performed. It was found that the overall survival of cluster1 was significantly lower than cluster2 (Fig. 5C).

### Establishment of the DCGs prognosis model based on the TCGA-LUAD training set

**Fig. 6:**
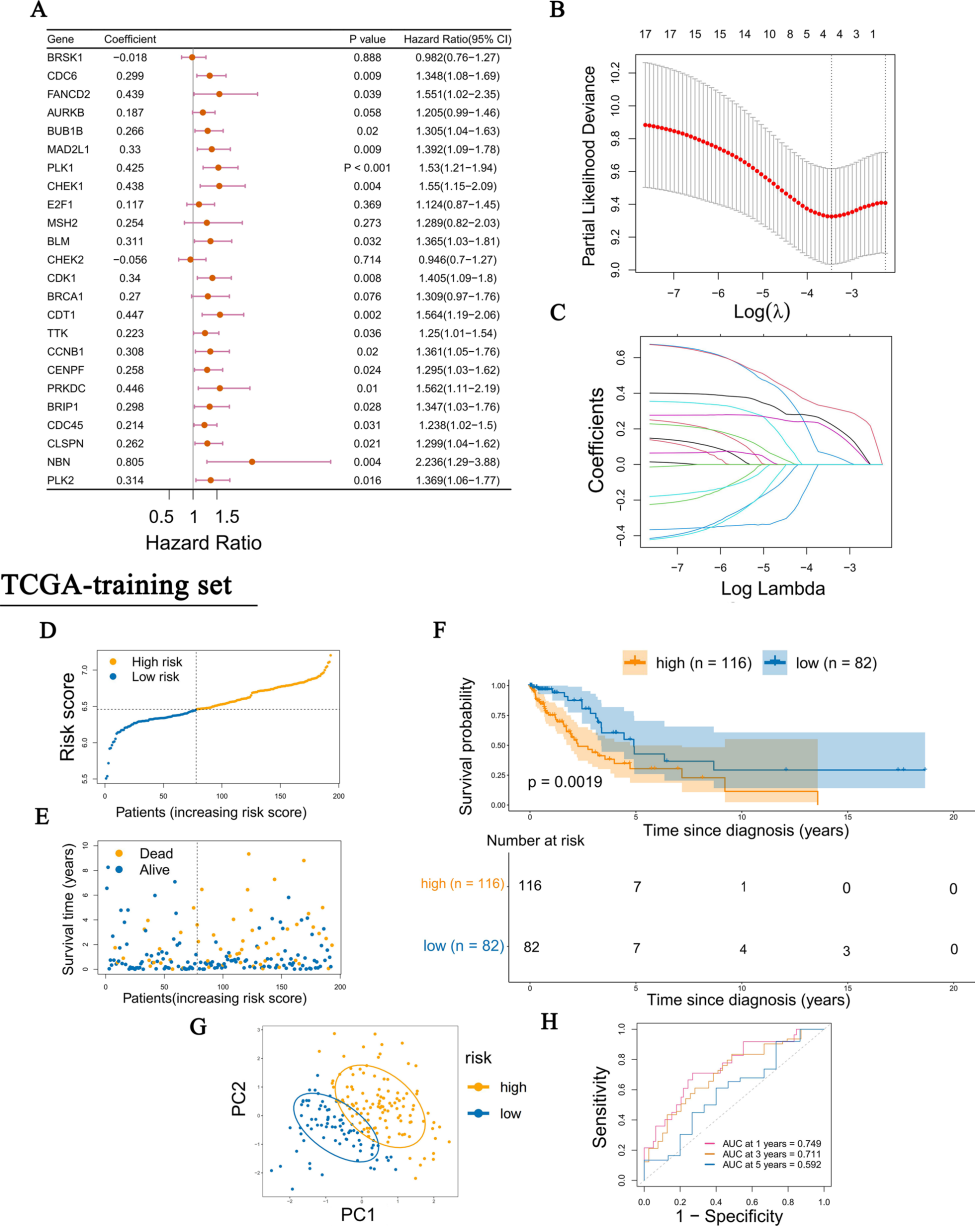
Prognosis model based on the DCGs signature. **A** A forest plot of univariate Cox regression analysis for 24 DCGs. **B** Cross-validation for optimal parameter selection in the LASSO regression. **C** Partial likelihood deviation under the number of different variables. **D** Distribution of patients based on the risk score. **E**, **F** Analysis of the survival rate and survival status in the two risk groups. **G** Principal component analysis (PCA) of the 4-DCGs signature. **H** The time-dependent receiver operating characteristic (ROC) of 4-DCGs signature

First, the univariate COX regression was used to screen DCGs affecting the prognosis in TCGA-LUAD training set patients (Fig. 6A). A total of 18 prognosis DCGs (*P* < 0.05, |HR|> 1) were selected into the LASSO regression model for further narrowing down the candidate genes, and a 4-DCGs signature was finally established based on the best λ value of 0.03165087 (Fig. 6B, C). The risk score was calculated as follows: Risk score = (0.054*PLK1exp.) + (0.199*PLK2exp.) + (0.229*PRKDCexp.) + (0.320*NBNexp.).

LUAD training set patients were divided into high-risk and low-risk groups according to the optimal cutoff value of the risk score (using the X-tile software, Fig. S2A, B in Additional file 2) (Fig. 6D). Principal component analysis (PCA) showed a clear distinction between the two risk groups (Fig. 6G). Subsequently, survival analysis revealed that patients in the high-risk group had poorer survival than the low-risk group (Fig. 6E, F). Finally, to assess the model’s predictive capability, we constructed the time-dependent receiver operating characteristic (ROC). The areas under the ROC curve (AUCs) at 1 year, 3 years, and 5 years were 0.749, 0.711, and 0.592 (Fig. 6H). Patients in the test set were also divided into high-risk and low-risk groups using the same cutoff value (6.46). The results of PCA and survival analysis were consistent with the training set. The AUCs of the test set at 1 year, 3 years, and 5 years were 0.698, 0.710, and 0.793 (Fig. S2C–G in Additional file 2).

### Validation of the 4-DCGs signature prognostic model

**Fig. 7:**
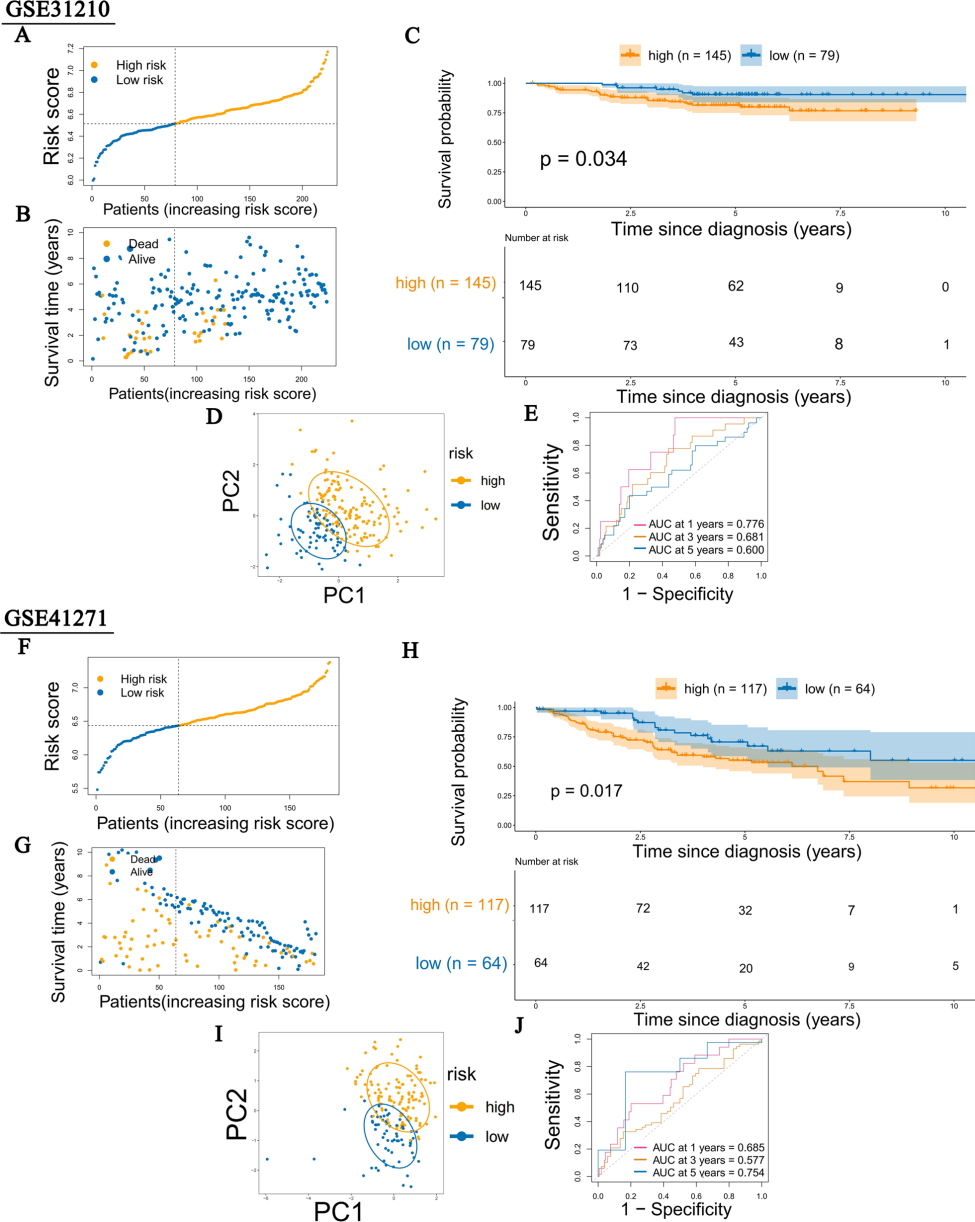
The validation of the DCGs signature Prognosis model in two GEO cohorts. **A**, **F** Distribution of patients based on the risk score. **B**, **C**, **G**, **H** Analysis of the survival rate and survival status in the two risk groups. **D**, **I** Principal component analysis (PCA) of the 4-DCGs signature. **E**, **J** The time-dependent receiver operating characteristic (ROC) of 4-DCGs signature

To further validate the reliability of the model, two independent GEO-LUAD cohorts, GSE31210 (n = 224) and GSE42171 (n = 181), were included as the validation cohorts. The patients in two validation cohorts were also divided into high-risk and low-risk groups according to the same cutoff value as in the TCGA training set (Fig. 7A, F). PCA analysis also displayed a clear distinction between the two groups (Fig. 7D, I). Consistent with the results in the TCGA cohort, the high-risk group patients had a poorer prognosis than the low-risk group patients in the two validation cohorts (Fig. 7B, C, G, H). Furthermore, the AUCs of the two validation cohorts displayed good predictive ability. The AUCs of GSE31210 at 1 year, 3 years, and 5 years were 0.776, 0.681, and 0.600 (Fig. 7E), and the AUCs of GSE42171 at 1 year, 3 years, and 5 years were 0.685, 0.664 and 0.754 (Fig. 7J). These results indicated that our 4-DCGs signature could reliably predict LUAD patient prognosis.

### Establishment of a prognosis nomogram combining risk score and clinical features for LUAD patients

**Fig. 8:**
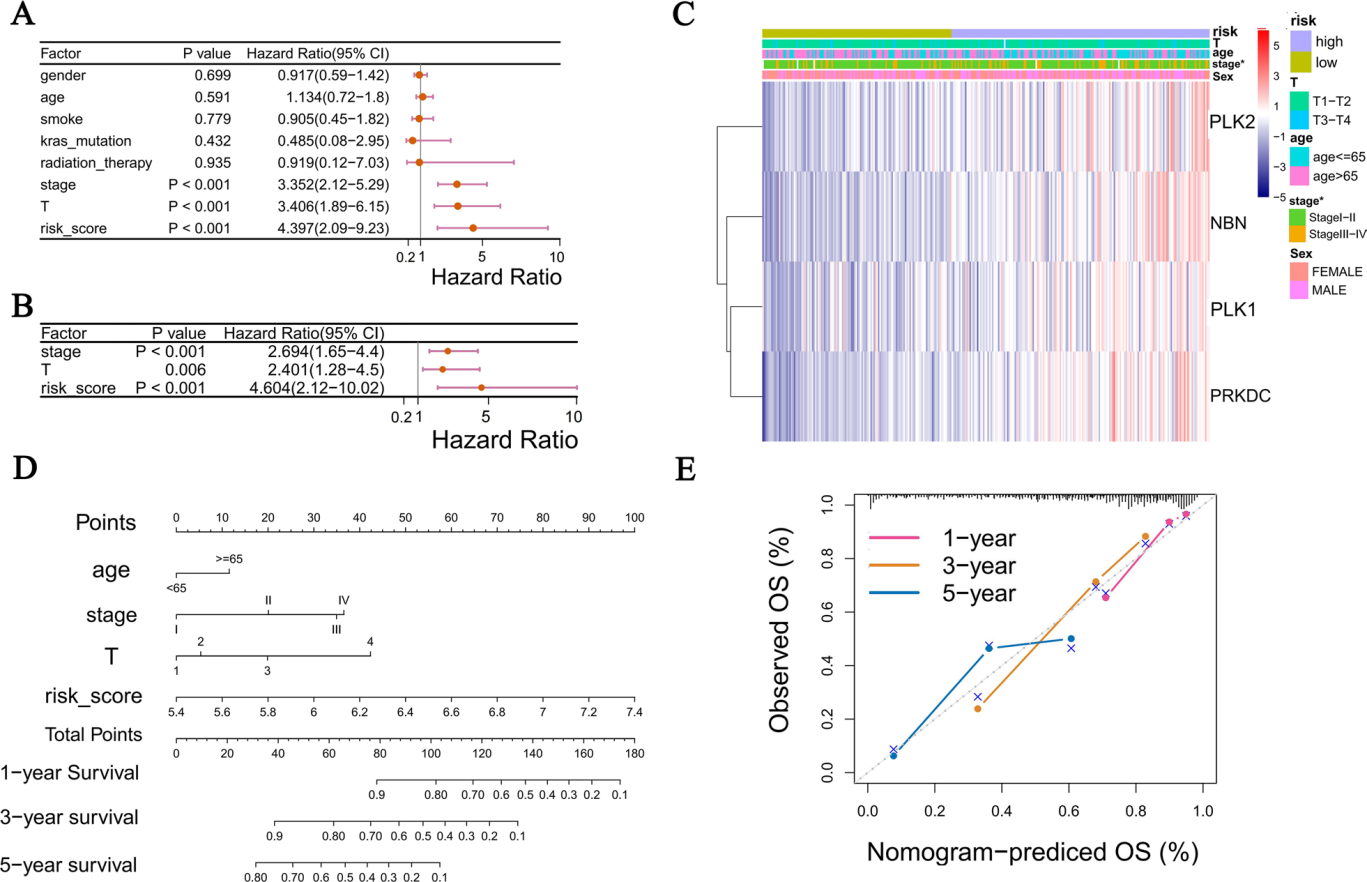
Establishment of a prognosis nomogram based on TCGA-LUAD. **A** Univariate COX regression analysis for the risk score and clinical characteristics. **B** Multivariate COX regression analysis for independent prognostic factors. **C** Heatmap for 4-DCGs and the correlation between clinical features and the risk groups (*P < 0.05). **D** Prognosis nomogram for predicting the survival of patients based on multivariate Cox regression analysis. **E** The calibration curves of the Nomogram for 1-year, 3-year, and 5-year survival in LUAD patients

To assess whether the 4-DCGs signature could be independent risk factors affecting LUAD prognosis, we performed univariate and multivariate COX regression analysis combining DCGs risk scores with clinical characteristics. Variables screened by the univariate COX regression (*P* < 0.05) were included in the multivariate COX regression (Fig. 8A). The risk score, stage, and T classification were determined as independent factors influencing LUAD prognosis (Fig. 8B). A clinicopathological information heatmap displayed that 4 DCGs were upregulated in the high-risk groups, and the stage had a significant difference in the two risk groups (*P* < 0.05) (Fig. 8C). Finally, to improve the clinical applicability of the model, a prognostic nomogram was constructed based on the results of the multivariate COX regression (Fig. 8D). Calibration curves, used to evaluate the prediction capability of the nomogram, indicated that the nomogram has a good prediction accuracy for the 1 year, 3 years, and 5 years survival (Fig. 8E).

### Functional enrichment of the 4-DCGs signature

**Fig. 9:**
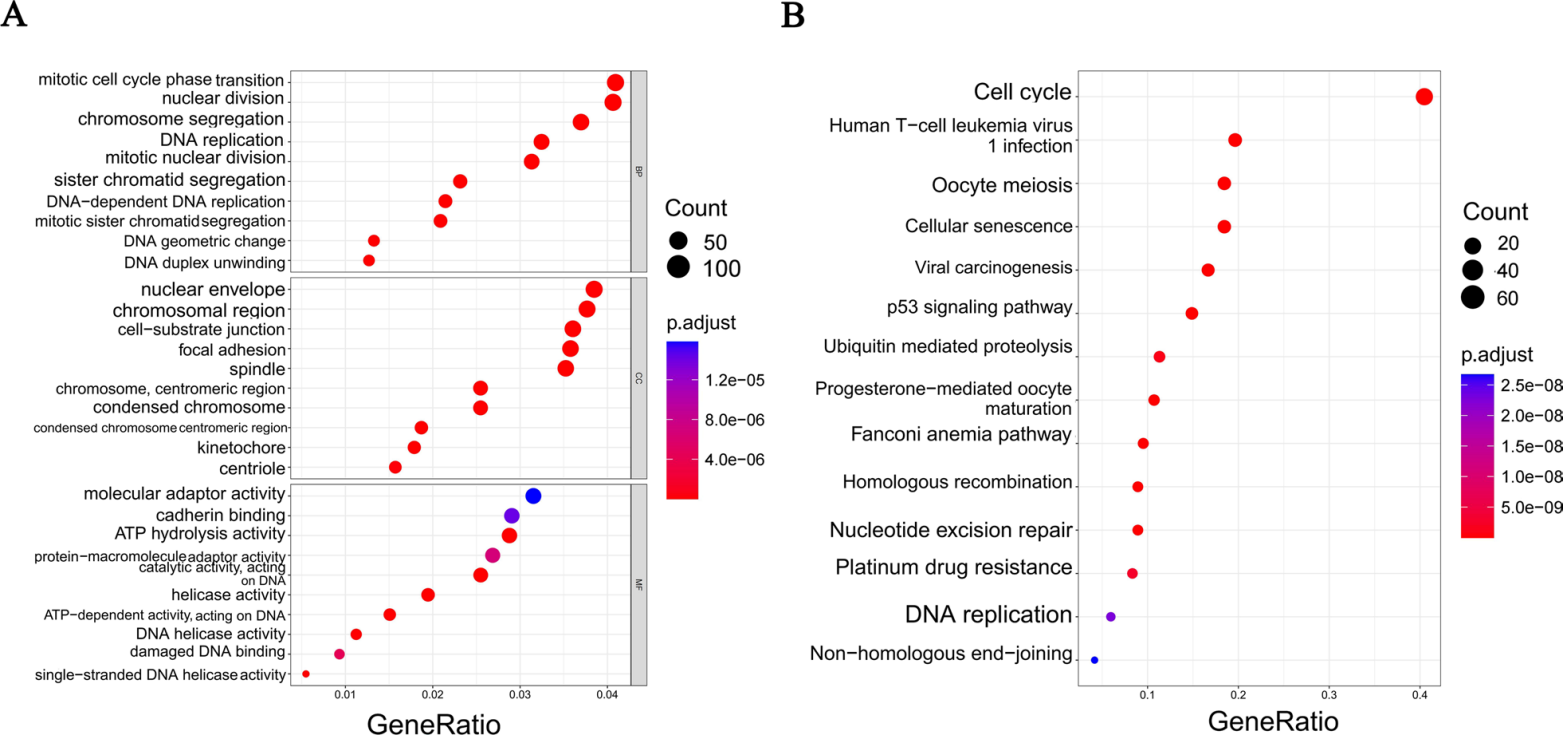
Functional enrichment of the 4-DCGs signature in TCGA-LUAD. **A** GO enrichment analysis of 4-DCGs, BP: Biological process; MF: Molecular function; CC: Cellular components. **B** KEGG enrichment analysis of 4-DCGs

To further investigate the underlying molecular heterogeneity in two risk groups based on the 4-DCGs signature, we performed a functional enrichment analysis of GO and KEGG for the DEGs between two risk groups in TCGA-LUAD. A total of 3882 DEGs were screened between the high-risk and low-risk groups (*P* < 0.05). The enrichment results showed that except enriched in DDR and cell cycle functions, these DEGs significantly enriched in immune and chemotherapy reactivity pathways, such as Human T cell leukemia virus 1 infection and platinum drug resistance (Fig. 9A, B).

### Comparison of immune activity in two risk groups

**Fig. 10:**
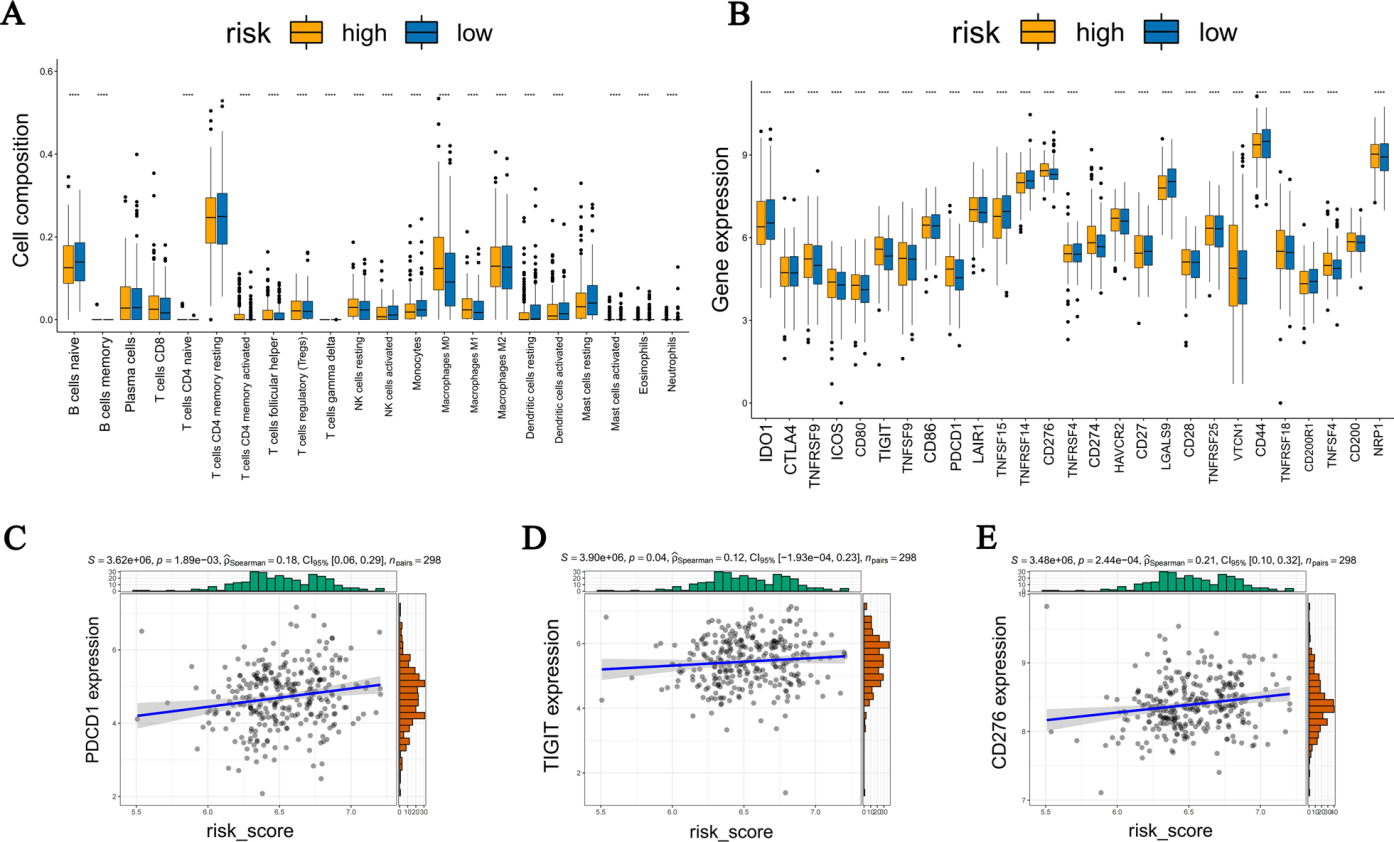
Immunoactivity analysis of two risk groups in TCGA-LUAD. **A** The composition of 22 types of tumor-infiltrating immune cells in the TCGA-LUAD samples. **B** The expression of 27 immune checkpoints in two risk groups. **C**–**E** Correlation analysis for the expression of immune checkpoints and risk scores

To explore the relationship between the 4 gene signature and LUAD immune microenvironment, we compared the composition of 22 tumor-infiltrating immune cells between high-risk and low-risk groups in TCGA-LUAD. The results indicated that the infiltration levels of most immune cells were lower in the high-risk group (Fig. 10A). The TIMER database results revealed DCGs closely correlated with immune cells (Fig. S3 in Additional file 2). In addition, the relationship between the expression of 27 immune checkpoints and two risk groups was further investigated. Most immune checkpoints were highly expressed in the high-risk group (Fig. 10B), and the expression levels of PDCD1, TIGIT, and CD276 were significantly correlated with the risk score (Fig. 10C–E). The results of validation cohorts are shown in Fig. S4 in Additional file 2.

### Comparison of chemoradiotherapy response in two risk groups

**Fig. 11:**
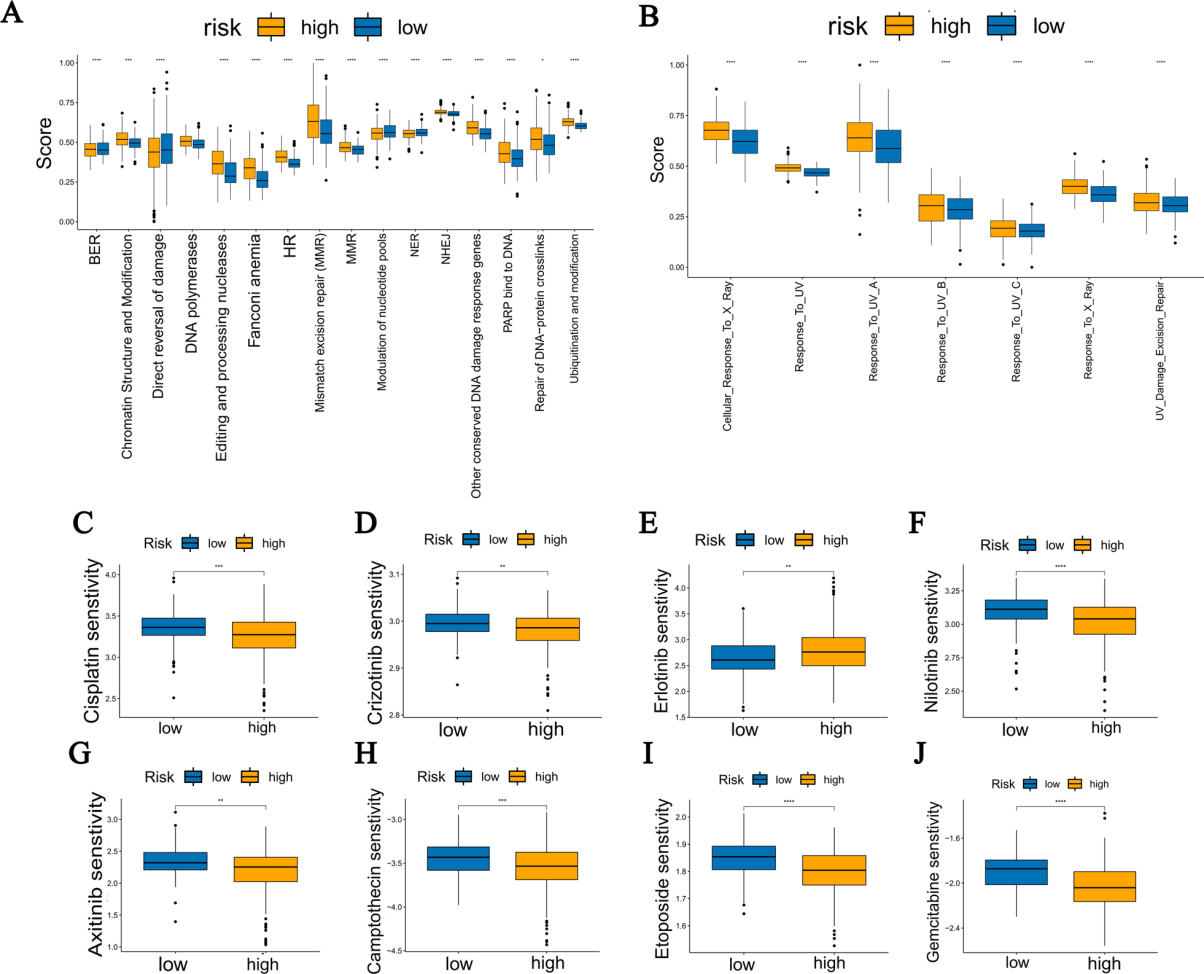
Comparison of chemoradiotherapy response in TCGA-LUAD. **A** The ssGSEA scores of 16 DDR pathways in the two risk groups. **B** The ssGSEA scores of X-ray and UV response in the two risk groups. **C**–**F** The sensitivity analysis of LUAD common chemotherapy agents (Cisplatin, Crizotinib, Erlotinib, and Nilotinib) in two risk groups. **G**–**J** The sensitivity analysis of other cancers’ common chemotherapy agents (Axitinib, Camptothecin, Etoposide, and Gemcitabine)

Radiotherapy and chemotherapy are the dominant treatments for cancer therapy. Therefore, we first compared the scores of 16 main DDR pathways, UV and X-ray responses in two groups using ssGSEA (single sample Gene Set Enrichment Analysis). Almost all DDR pathways were upregulated in the high-risk group (Fig. 11A), and there was higher reactivity for UV and X-ray in the high-risk group (Fig. 11B), indicating that patients in the high-risk group had the stronger radiation resistance.

Furthermore, the sensitivity of 4 common lung adenocarcinoma chemotherapy agents (Cisplatin, Crizotinib, Erlotinib, and Nilotinib) and 6 other tumor chemotherapy agents (Axitinib, Camptothecin, Etoposide, and Gemcitabine) for two risk groups were analyzed based on Genomics of Drug Sensitivity of Cancer (GDSC) using the R package “pRRophetic”. Results displayed that besides Nilotinib, the high-risk group had lower sensitivity to all other drugs (Fig. 11C–J), which meant that high-risk patients were more insensitive to chemotherapy. The results of validation cohorts are shown in Fig. S5 in Additional file 2**.** Furthermore, the DEGs of two risk groups were imported into the CMap database to discover potential small molecule drugs for treatment. The top 15 positively correlated molecules and the top 15 negatively correlated molecules were obtained from the CMap (Table S4 in Additional file 1). The patients in the low-risk group could benefit from the positively correlated molecules.

## Discussion

In recent years, the association between some molecular markers and the prognosis of LUAD has been found in a great number of studies. For example, Koh et al. found that PD-1 overexpression in patients with LUAD resulted in poor overall survival and progression-free survival [29]. Wang et al. indicated that the decreased expression of miR-133a in patients with LUAD was related to poor prognosis [30]. Takamizawa et al. found that the overexpression of let-7 microRNA in postoperative patients with LUAD was associated with a relatively short survival time [31]. However, due to the high heterogeneity of cancer, single-function genes explaining the patient prognosis may be farfetched. Furthermore, some problems hamper the prediction accuracy of these prognostic signatures, such as insufficient clinical samples and a lack of external independent validation.

The cell cycle checkpoints play an essential role in DDR. Moreover, it and DDR are respectively related to LUAD prognosis. Therefore, we sought to select genes with both cell cycle checkpoints and DDR functions in this study, developed a prognosis model in the TCGA-LUAD cohort, and validated it in two independent GEO cohorts. To make the results more scientific and reliable, the TCGA cohort was divided into a training set and a test set according to the 2:1 ratio. The training set was used to construct the model, the test set was used to test the model’s efficacy, and the independent GEO cohorts were finally used to verify the model. The LASSO regression, screening for the key variables, has been widely used to construct prognosis models for different tumors, and the patient classification based on risk score, which is calculated by the LASSO coefficient, has feasible clinical guiding significance [32–34]. Thus, we combined univariate COX regression and LASSO regression to establish a 4-DCGs prognostic model with high predictive accuracy for LUAD survival, especially in 3-year survival (AUC = 0.711, 0.710). Greater AUC indicates a higher diagnostic value of the test, and AUC greater than 0.7 means high test accuracy. From this, the model we constructed has some clinical application value. In addition, to improve the clinical applicability, we also identified independent prognostic factors combined with the patient's clinical features to establish a prognostic nomogram with good predictive power. For example, a 70-year-old LUAD patient with stage II, T3, and risk score (4-DCGs signature) equal to 6.8 would score a total of 122 points (12 points for age, 20 points for stage, 20 points for T classification, and 70 points for risk score). For this patient, the predicted survival for 1 year, 3 years, and 5 years was 63.0%, 19%, and < 10.0%, respectively.

The 4-DCGs signature consists of PLK1, PLK2, PRKDC, and NBN. PLK1 (Polo-like kinase 1), a member of the polo family of serine/threonine protein kinases, is an essential regulator of cell cycle progression that induces activation of DNA damage checkpoint [35, 36]. The overexpression of PLK1 appeared in various cancers with poor prognosis and survival. In addition, studies have shown that inhibition of PLK1 promotes tumor cell apoptosis in lung cancer [37, 38]. PLK2, also called SNK, regulates the replication of centrosomes during cell division and can be induced by P53 to activate the G2 checkpoints in the DNA damage response [39]. It was reported that PLK2 promotes tumor growth by targeting the FBXW7/ Cyclin E pathway [40, 41]. The PRKDC gene encodes DNA-PK protein kinase, a protein kinase required for cell cycle progression during mitosis and the NHEJ pathway. Moreover, PRKDC can be a drug target for immune checkpoint inhibitors, while the inhibition of DNA-PK can also enhance the chemosensitivity and radiosensitivity of NSCLC. NBN, encoding the Nibrin protein, is a component of the Mre11-Rad50-Nbs (MRN) complex, which can trigger cell cycle checkpoint activation through interaction with ATM proteins, and plays a vital role in the DDR [42]. Increased expression of NBN genes in breast and ovarian cancer cells can induce chemoresistance and poor prognosis, and mutations in NBN can also cause Nijmegen breakage syndrome (NBS), leading to low immune function and abnormal lymphocyte function in patients [43, 44]. To sum up, consistent with our results, high expression of 4-DCGs was closely associated with cancer progression and poor prognosis, and also suggested that these four genes may influence the immune environment and chemoradiation resistance of LUAD patients.

Increasing evidence suggests that tumor development and progression largely depend on the complex microenvironment in which they reside, including the tumor cells and their surrounding immune cells [45]. Therefore, to further explore the potential prognostic mechanism of risk grouping based on the 4-DCGs signature model, we compared the compositions of the 22 tumor-infiltrating immune cells in the two risk groups. The results showed that infiltration levels of naive B cells, activated NK cells, monocytes, and activated dendritic cells were significantly lower in the high-risk group. In contrast, M0 macrophages and resting NK cells were higher in the high-risk group. Naive B cells are a type of lymphocyte, and many studies have reported that its infiltration level correlated with a favorable prognosis in NSCLC [46, 47]. NK cells involved in tumor immunity can be divided into resting and activated subtypes [48]. Generally, the higher the proportion of resting NK cells or the lower the proportion of activated NK cells is, the higher the level of tumor infiltration will be, which favors the formation of the tumor microenvironment [49]. Recruitment of monocytes in the early stages of tumor progression can be found in multiple types of cancer, where monocytes directly kill malignant cells by cytokine-mediated cell death and phagocytosis [50]. An emerging study indicated that the anti-tumor effect of dendritic cells (DCs) could be reduced by the low DCs count inducing the low antigen presentation efficiency of tumor-invasive DCs [51]. Tumor-associated macrophages (TAMs) function as a promoter during tumor progression. TAM consists of several macrophages’ phenotypes, including M0 (inactivated macrophages), M1 (classical activated), and M2 (alternately activated). M2 cells are polarized from M0 macrophages and promote immunosuppression and angiogenesis by producing immunosuppressive factors, interleukin-10 [52, 53]. It follows then that our high-risk group with low anti-tumor immune cells and high pro-tumor immune cells showed a low tumor-suppressive immune microenvironment. In other words, our 4-DCGs signature could somewhat predict the immune activity of LUAD patients.

Furthermore, in our study, the immune checkpoint expression levels were generally elevated in the high-risk group, and immune checkpoints (PDCD1, TIGIT, and CD276) expression were positively correlated with the risk scores. Immune activation can be increased by blocking immune checkpoints. Thus immune checkpoint gene expression is considered an indicator of immunotherapy response in clinical practice. Blocking the immune checkpoint has become a novel approach to eliminating the immunosuppressive microenvironment to enable tumor immunotherapy [54, 55]. Lower immunoactivities’ cell infiltration and higher immune checkpoint expression may explain why a poor prognosis appeared in high-risk LUAD patients. Targeted immune checkpoints, in turn, may be a viable option for immunotherapy of the high-risk group.

Radioresistance in cancer cells remains the main limitation in radiotherapy applications. DNA double-strand breaks are the most lethal damage caused by ionizing radiation and trigger a series of DNA damage responses (DDRs) that help cells recover from radiation damage. These DDRs confer radioresistance to the tumor, bringing a poor prognosis [56]. The 4-DCGs have DDR function, therefore we compared the ssGSEA scores of the DDR pathway, X-ray, and UV response in different risk groups. The results displayed that almost all DDR pathways, X-ray, and UV responses were upregulated in the high-risk group, indicating the high radioresistance may contribute to a poor prognosis in patients of the high-risk group. It also suggested that the 4-DCGs can predict radiotherapy responsiveness in LUAD patients.

Platinum-based chemotherapy is the cornerstone of treatment for LUAD patients. However, many patients relapse because of resistance to tumor-killing drugs, leading to a poor prognosis [57, 58]. Therefore, the distinction of primary-resistant LUAD patient populations can maximize the clinical benefit of these patients [59]. Our functional enrichment results showed that the differential genes in the two risk groups were significantly enriched in pathways with platinum drug resistance, thus speculating that our risk grouping could predict drug sensitivity. Unsurprisingly, sensitivity analysis of common anti-tumor drugs displayed that the high-risk group showed low sensitivity in the common anti-lung adenocarcinoma drugs (Cisplatin, Crizotinib, Nilotinib) and other anti-tumor drugs, indicating that the low drug sensitivity contributes to a poor prognosis in the high-risk group. In other words, our grouping could distinguish drug-sensitive populations to some extent. We found that Erlotinib had high sensitivity in the high-risk group, suggesting that the treatment for the high-risk group could adopt Erlotinib. In addition, we also predicted relevant small molecule drug targets using the drug analysis database, which offers some references for the clinical treatment of risk grouping.

In conclusion, the 4-DCGs signature participates in the DNA damage repair and cell cycle checkpoints regulation, and its higher expression implies the better repair in damaged cells of high-risk group patients after chemoradiotherapy, meaning the higher chemoradioresistence. Therefore, combined with the immune activity analysis, the prognosis model constructed by the 4-DCGs signature could reasonably predict the prognosis of LUAD patients. Our study provides auxiliary guidance for the clinical therapy of LUAD, while more clinical cohorts and experiments are required to validate these results further.

## Conclusions

The prognosis model based on the 4-DCGs signature could well predict the survival rate, immune activity, and chemoradiotherapy responsiveness of LUAD patients. This study provides some guidance for the treatment and prognosis evaluation of LUAD.

## Supplementary Information

**Additional file 1.** Additional figures.

**Additional file 2.** Additional tables.

## Data Availability

Public datasets this study used can be found in The Cancer Genome Atlas (https://portal.gdc.cancer.gov/) and the Gene Expression Omnibus (https://www.ncbi.nlm.nih.gov/geo/). Additional data named “Additional file 1” and “Additional file 2” have been uploaded.

## Abbreviations

LUAD: Lung adenocarcinoma
DDR: DNA damage repair
DCGs: DDR-related genes with cell cycle checkpoint function
LASSO: Least absolute shrinkage and selection operator
OS: Overall survival
GO: Ontology
KEGG: Kyoto Encyclopedia of Genes and Genomes
DEGs: Differentially expressed genes
PPI: Protein–protein interaction
ROC: The receiver operator characteristic
AUCs: The areas under the ROC curve
PCA: Principal component analysis
ssGSEA: Single-sample gene set enrichment analysis
DCs: Dendritic cells
TAMs: Tumor-associated macrophages
M0: Inactivated macrophages
M1: Classical activated
M2: Alternately activated
DDRs: DNA damage responses
