# Supplementary material for: The DDR-related gene signature with cell cycle checkpoint function predicts prognosis, immune activity, and chemoradiotherapy response in lung adenocarcinoma
Source: Respir Res. 2022 Jul 15;23:190. doi: 10.1186/s12931-022-02110-w (PMC9288070; doi:10.1186/s12931-022-02110-w)
Supplement: Supplementary file 1 — Additional file 1. Additional figures. [file 12931_2022_2110_MOESM1_ESM.docx]

**Additional file 1: Table S1** Details of 296 DDR genes.

| **Gene symbol** | **Full name** | **Ensemble ID** |
| --- | --- | --- |
| YWHAB | Tyrosine 3-Monooxygenase | ENSG00000166913 |
| TFAP2C | Transcription Factor AP-2 Gamma | ENSG00000087510 |
| ATRIP | ATR Interacting Protein | [ENSG00000164053](https://www.ensembl.org/Homo_sapiens/geneview?gene=ENSG00000164053) |
| BRSK1 | BR Serine/Threonine Kinase 1 | [ENSG00000160469](https://www.ensembl.org/Homo_sapiens/geneview?gene=ENSG00000160469) |
| CCNE2 | Cyclin E2 | [ENSG00000175305](https://www.ensembl.org/Homo_sapiens/geneview?gene=ENSG00000175305) |
| CDC6 | Cell Division Cycle 6 | [ENSG00000094804](https://www.ensembl.org/Homo_sapiens/geneview?gene=ENSG00000094804) |
| CDK5 | Cyclin Dependent Kinase 5 | [ENSG00000164885](https://www.ensembl.org/Homo_sapiens/geneview?gene=ENSG00000164885) |
| CENPT | Centromere Protein T | [ENSG00000102901](https://www.ensembl.org/Homo_sapiens/geneview?gene=ENSG00000102901) |
| COPS5 | COP9 Signalosome Subunit 5 | [ENSG00000121022](https://www.ensembl.org/Homo_sapiens/geneview?gene=ENSG00000121022) |
| DTX3L | Deltex E3 Ubiquitin Ligase 3L | [ENSG00000163840](https://www.ensembl.org/Homo_sapiens/geneview?gene=ENSG00000163840) |
| FANCD2 | FA Complementation Group D2 | [ENSG00000144554](https://www.ensembl.org/Homo_sapiens/geneview?gene=ENSG00000144554) |
| HELQ | Helicase, POLQ Like | ENSG00000163312 |
| LATS2 | Large Tumor Suppressor Kinase 2 | [ENSG00000150457](https://www.ensembl.org/Homo_sapiens/geneview?gene=ENSG00000150457) |
| MIS18A | MIS18 Kinetochore Protein A | [ENSG00000159055](https://www.ensembl.org/Homo_sapiens/geneview?gene=ENSG00000159055) |
| NEK7 | NIMA Related Kinase 7 | [ENSG00000151414](https://www.ensembl.org/Homo_sapiens/geneview?gene=ENSG00000151414) |
| CDKN1B | Cyclin Dependent Kinase Inhibitor 1B | [ENSG00000111276](https://www.ensembl.org/Homo_sapiens/geneview?gene=ENSG00000111276) |
| ERCC6L | ERCC Excision Repair 6 Like | [ENSG00000186871](https://www.ensembl.org/Homo_sapiens/geneview?gene=ENSG00000186871) |
| PPP2R1A | Protein Phosphatase 2 Scaffold Subunit Aalpha | [ENSG00000105568](https://www.ensembl.org/Homo_sapiens/geneview?gene=ENSG00000105568) |
| RAD23B | RAD23 Homolog B, Nucleotide Excision Repair Protein | [ENSG00000119318](https://www.ensembl.org/Homo_sapiens/geneview?gene=ENSG00000119318) |
| RECQL4 | RecQ Like Helicase 4 | [ENSG00000160957](https://www.ensembl.org/Homo_sapiens/geneview?gene=ENSG00000160957) |
| SLK | STE20 Like Kinase | [ENSG00000065613](https://www.ensembl.org/Homo_sapiens/geneview?gene=ENSG00000065613) |
| RCC2 | Regulator Of Chromosome Condensation 2 | [ENSG00000179051](https://www.ensembl.org/Homo_sapiens/geneview?gene=ENSG00000179051) |
| TPT1 | Tumor Protein, Translationally-Controlled 1 | [ENSG00000133112](https://www.ensembl.org/Homo_sapiens/geneview?gene=ENSG00000133112) |
| UBE2S | Ubiquitin Conjugating Enzyme E2 S | [ENSG00000108106](https://www.ensembl.org/Homo_sapiens/geneview?gene=ENSG00000108106) |
| WEE1 | WEE1 G2 Checkpoint Kinase | [ENSG00000166483](https://www.ensembl.org/Homo_sapiens/geneview?gene=ENSG00000166483) |
| YWHAE | Tyrosine 3-Monooxygenase/Tryptophan 5-Monooxygenase | [ENSG00000108953](https://www.ensembl.org/Homo_sapiens/geneview?gene=ENSG00000108953) |
| ANAPC1 | Anaphase Promoting Complex Subunit 1 | [ENSG00000153107](https://www.ensembl.org/Homo_sapiens/geneview?gene=ENSG00000153107) |
| AURKA | Aurora Kinase A | [ENSG00000087586](https://www.ensembl.org/Homo_sapiens/geneview?gene=ENSG00000087586) |
| BRSK2 | BR Serine/Threonine Kinase 2 | [ENSG00000174672](https://www.ensembl.org/Homo_sapiens/geneview?gene=ENSG00000174672) |
| CCNF | Cyclin F | [ENSG00000162063](https://www.ensembl.org/Homo_sapiens/geneview?gene=ENSG00000162063) |
| CDC7 | Cell Division Cycle 7 | [ENSG00000097046](https://www.ensembl.org/Homo_sapiens/geneview?gene=ENSG00000097046) |
| CDK6 | Cyclin Dependent Kinase 6 | [ENSG00000105810](https://www.ensembl.org/Homo_sapiens/geneview?gene=ENSG00000105810) |
| CEP55 | Centrosomal Protein 55 | [ENSG00000138180](https://www.ensembl.org/Homo_sapiens/geneview?gene=ENSG00000138180) |
| CCP110 | Centriolar Coiled-Coil Protein 110 | ENSG00000103540 |
| DCTN1 | Dynactin Subunit 1 | [ENSG00000204843](https://www.ensembl.org/Homo_sapiens/geneview?gene=ENSG00000204843) |
| FEN1 | Flap Structure-Specific Endonuclease 1 | [ENSG00000168496](https://www.ensembl.org/Homo_sapiens/geneview?gene=ENSG00000168496) |
| HIPK2 | Homeodomain Interacting Protein Kinase 2 | [ENSG00000064393](https://www.ensembl.org/Homo_sapiens/geneview?gene=ENSG00000064393) |
| LIG4 | DNA Ligase 4 | [ENSG00000174405](https://www.ensembl.org/Homo_sapiens/geneview?gene=ENSG00000174405) |
| MLH1 | MutL Homolog 1 | [ENSG00000076242](https://www.ensembl.org/Homo_sapiens/geneview?gene=ENSG00000076242) |
| NHEJ1 | Non-Homologous End Joining Factor 1 | [ENSG00000187736](https://www.ensembl.org/Homo_sapiens/geneview?gene=ENSG00000187736) |
| TP53 | Tumor Protein P53 | [ENSG00000141510](https://www.ensembl.org/Homo_sapiens/geneview?gene=ENSG00000141510) |
| PIN1 | Peptidylprolyl Cis/Trans Isomerase, NIMA-Interacting 1 | [ENSG00000127445](https://www.ensembl.org/Homo_sapiens/geneview?gene=ENSG00000127445) |
| PPP2R2A | Protein Phosphatase 2 Regulatory Subunit Balpha | [ENSG00000221914](https://www.ensembl.org/Homo_sapiens/geneview?gene=ENSG00000221914) |
| RAD50 | RAD50 Double Strand Break Repair Protein | [ENSG00000113522](https://www.ensembl.org/Homo_sapiens/geneview?gene=ENSG00000113522) |
| RECQL5 | RecQ Like Helicase 5 | [ENSG00000108469](https://www.ensembl.org/Homo_sapiens/geneview?gene=ENSG00000108469) |
| SMARCA4 | Regulator Of Chromatin, Subfamily A, Member 4 | [ENSG00000127616](https://www.ensembl.org/Homo_sapiens/geneview?gene=ENSG00000127616) |
| TDP1 | Tyrosyl-DNA Phosphodiesterase 1 | [ENSG00000042088](https://www.ensembl.org/Homo_sapiens/geneview?gene=ENSG00000042088) |
| TPX2 | TPX2 Microtubule Nucleation Factor | [ENSG00000088325](https://www.ensembl.org/Homo_sapiens/geneview?gene=ENSG00000088325) |
| UBE2T | Ubiquitin Conjugating Enzyme E2 T | [ENSG00000077152](https://www.ensembl.org/Homo_sapiens/geneview?gene=ENSG00000077152) |
| WRN | WRN RecQ Like Helicase | [ENSG00000165392](https://www.ensembl.org/Homo_sapiens/geneview?gene=ENSG00000165392) |
| YWHAH | Tyrosine 3-Monooxygenase/Tryptophan 5-Monooxygenase Activation Protein Eta | [ENSG00000128245](https://www.ensembl.org/Homo_sapiens/geneview?gene=ENSG00000128245) |
| ANAPC10 | Anaphase Promoting Complex Subunit 10 | [ENSG00000164162](https://www.ensembl.org/Homo_sapiens/geneview?gene=ENSG00000164162) |
| AURKB | Aurora Kinase B | [ENSG00000178999](https://www.ensembl.org/Homo_sapiens/geneview?gene=ENSG00000178999) |
| BUB1B | BUB1 Mitotic Checkpoint Serine/Threonine Kinase B | [ENSG00000156970](https://www.ensembl.org/Homo_sapiens/geneview?gene=ENSG00000156970) |
| CCNH | Cyclin H | [ENSG00000134480](https://www.ensembl.org/Homo_sapiens/geneview?gene=ENSG00000134480) |
| CDC73 | Cell Division Cycle 73 | [ENSG00000134371](https://www.ensembl.org/Homo_sapiens/geneview?gene=ENSG00000134371) |
| CDK7 | Cyclin Dependent Kinase 7 | [ENSG00000134058](https://www.ensembl.org/Homo_sapiens/geneview?gene=ENSG00000134058) |
| CHFR | Checkpoint With Forkhead And Ring Finger Domains | [ENSG00000072609](https://www.ensembl.org/Homo_sapiens/geneview?gene=ENSG00000072609) |
| RBBP8 | RB Binding Protein 8, Endonuclease | [ENSG00000101773](https://www.ensembl.org/Homo_sapiens/geneview?gene=ENSG00000101773) |
| DYRK2 | Dual Specificity Tyrosine Phosphorylation Regulated Kinase 2 | [ENSG00000127334](https://www.ensembl.org/Homo_sapiens/geneview?gene=ENSG00000127334) |
| FOS | Fos Proto-Oncogene, AP-1 Transcription Factor Subunit | [ENSG00000170345](https://www.ensembl.org/Homo_sapiens/geneview?gene=ENSG00000170345) |
| HJURP | Holliday Junction Recognition Protein | [ENSG00000123485](https://www.ensembl.org/Homo_sapiens/geneview?gene=ENSG00000123485) |
| MAD2L1 | Mitotic Arrest Deficient 2 Like 1 | ENSG00000164109 |
| MRE11 | MRE11 Homolog, Double Strand Break Repair Nuclease | [ENSG00000020922](https://www.ensembl.org/Homo_sapiens/geneview?gene=ENSG00000020922) |
| ZC3HC1 | Zinc Finger C3HC-Type Containing 1 | [ENSG00000091732](https://www.ensembl.org/Homo_sapiens/geneview?gene=ENSG00000091732) |
| CDKN1C | Cyclin Dependent Kinase Inhibitor 1C | [ENSG00000129757](https://www.ensembl.org/Homo_sapiens/geneview?gene=ENSG00000129757) |
| PLK1 | Polo Like Kinase 1 | [ENSG00000166851](https://www.ensembl.org/Homo_sapiens/geneview?gene=ENSG00000166851) |
| PTPA | Protein Phosphatase 2 Phosphatase Activator | [ENSG00000119383](https://www.ensembl.org/Homo_sapiens/geneview?gene=ENSG00000119383) |
| RAD51 | RAD51 Recombinase | [ENSG00000051180](https://www.ensembl.org/Homo_sapiens/geneview?gene=ENSG00000051180) |
| RPA2 | Replication Protein A2 | [ENSG00000117748](https://www.ensembl.org/Homo_sapiens/geneview?gene=ENSG00000117748) |
| SMARCA5 | Regulator Of Chromatin, Subfamily A, Member 5 | [ENSG00000153147](https://www.ensembl.org/Homo_sapiens/geneview?gene=ENSG00000153147) |
| TERF2IP | TERF2 Interacting Protein | [ENSG00000166848](https://www.ensembl.org/Homo_sapiens/geneview?gene=ENSG00000166848) |
| TRIB2 | Tribbles Pseudokinase 2 | [ENSG00000071575](https://www.ensembl.org/Homo_sapiens/geneview?gene=ENSG00000071575) |
| USP1 | Ubiquitin Specific Peptidase 1 | ENSG00000162607 |
| WWOX | WW Domain Containing Oxidoreductase | [ENSG00000186153](https://www.ensembl.org/Homo_sapiens/geneview?gene=ENSG00000186153) |
| YWHAG | Tyrosine 3-Monooxygenase/Tryptophan 5-Monooxygenase | [ENSG00000170027](https://www.ensembl.org/Homo_sapiens/geneview?gene=ENSG00000170027) |
| ANAPC11 | Anaphase Promoting Complex Subunit 11 | [ENSG00000141552](https://www.ensembl.org/Homo_sapiens/geneview?gene=ENSG00000141552) |
| AURKC | Aurora Kinase C | [ENSG00000105146](https://www.ensembl.org/Homo_sapiens/geneview?gene=ENSG00000105146) |
| BUB3 | BUB3 Mitotic Checkpoint Protein | [ENSG00000154473](https://www.ensembl.org/Homo_sapiens/geneview?gene=ENSG00000154473) |
| CDC16 | Cell Division Cycle 16 | [ENSG00000130177](https://www.ensembl.org/Homo_sapiens/geneview?gene=ENSG00000130177) |
| CDCA2 | Cell Division Cycle Associated 2 | [ENSG00000184661](https://www.ensembl.org/Homo_sapiens/geneview?gene=ENSG00000184661) |
| CDK8 | Cyclin Dependent Kinase 8 | [ENSG00000132964](https://www.ensembl.org/Homo_sapiens/geneview?gene=ENSG00000132964) |
| CHEK1 | Checkpoint Kinase 1 | [ENSG00000149554](https://www.ensembl.org/Homo_sapiens/geneview?gene=ENSG00000149554) |
| CUEDC2 | CUE Domain Containing 2 | [ENSG00000107874](https://www.ensembl.org/Homo_sapiens/geneview?gene=ENSG00000107874) |
| E2F1 | E2F Transcription Factor 1 | [ENSG00000101412](https://www.ensembl.org/Homo_sapiens/geneview?gene=ENSG00000101412) |
| FOSB | FosB Proto-Oncogene, AP-1 Transcription Factor Subunit | [ENSG00000125740](https://www.ensembl.org/Homo_sapiens/geneview?gene=ENSG00000125740) |
| HUS1 | HUS1 Checkpoint Clamp Component | [ENSG00000136273](https://www.ensembl.org/Homo_sapiens/geneview?gene=ENSG00000136273) |
| MASTL | Microtubule Associated Serine/Threonine Kinase Like | [ENSG00000120539](https://www.ensembl.org/Homo_sapiens/geneview?gene=ENSG00000120539) |
| MSH2 | MutS Homolog 2 | [ENSG00000095002](https://www.ensembl.org/Homo_sapiens/geneview?gene=ENSG00000095002) |
| NPM1 | Nucleophosmin 1 | [ENSG00000181163](https://www.ensembl.org/Homo_sapiens/geneview?gene=ENSG00000181163) |
| TP63 | Tumor Protein P63 | [ENSG00000073282](https://www.ensembl.org/Homo_sapiens/geneview?gene=ENSG00000073282) |
| PLK2 | Polo Like Kinase 2 | [ENSG00000145632](https://www.ensembl.org/Homo_sapiens/geneview?gene=ENSG00000145632) |
| PPP5C | Protein Phosphatase 5 Catalytic Subunit | [ENSG00000011485](https://www.ensembl.org/Homo_sapiens/geneview?gene=ENSG00000011485) |
| RAD54L | RAD54 Like | [ENSG00000085999](https://www.ensembl.org/Homo_sapiens/geneview?gene=ENSG00000085999) |
| RIF1 | Replication Timing Regulatory Factor 1 | [ENSG00000080345](https://www.ensembl.org/Homo_sapiens/geneview?gene=ENSG00000080345) |
| SMARCAL1 | Regulator Of Chromatin, Subfamily A Like 1 | [ENSG00000138375](https://www.ensembl.org/Homo_sapiens/geneview?gene=ENSG00000138375) |
| TFCP2 | Transcription Factor CP2 | [ENSG00000135457](https://www.ensembl.org/Homo_sapiens/geneview?gene=ENSG00000135457) |
| TERF2 | Telomeric Repeat Binding Factor 2 | [ENSG00000132604](https://www.ensembl.org/Homo_sapiens/geneview?gene=ENSG00000132604) |
| USP10 | Ubiquitin Specific Peptidase 10 | [ENSG00000103194](https://www.ensembl.org/Homo_sapiens/geneview?gene=ENSG00000103194) |
| XPA | XPA, DNA Damage Recognition And Repair Factor | [ENSG00000136936](https://www.ensembl.org/Homo_sapiens/geneview?gene=ENSG00000136936) |
| SFN | Stratifin | [ENSG00000175793](https://www.ensembl.org/Homo_sapiens/geneview?gene=ENSG00000175793) |
| ANAPC2 | Anaphase Promoting Complex Subunit 2 | [ENSG00000176248](https://www.ensembl.org/Homo_sapiens/geneview?gene=ENSG00000176248) |
| BLM | BLM RecQ Like Helicase | [ENSG00000197299](https://www.ensembl.org/Homo_sapiens/geneview?gene=ENSG00000197299) |
| CABIN1 | Calcineurin Binding Protein 1 | [ENSG00000099991](https://www.ensembl.org/Homo_sapiens/geneview?gene=ENSG00000099991) |
| CDC20 | Cell Division Cycle 20 | [ENSG00000117399](https://www.ensembl.org/Homo_sapiens/geneview?gene=ENSG00000117399) |
| CDIP1 | Cell Death Inducing P53 Target 1 | [ENSG00000089486](https://www.ensembl.org/Homo_sapiens/geneview?gene=ENSG00000089486) |
| CDK9 | Cyclin Dependent Kinase 9 | [ENSG00000136807](https://www.ensembl.org/Homo_sapiens/geneview?gene=ENSG00000136807) |
| CHEK2 | Checkpoint Kinase 2 | [ENSG00000183765](https://www.ensembl.org/Homo_sapiens/geneview?gene=ENSG00000183765) |
| CCAR2 | Cell Cycle And Apoptosis Regulator 2 | [ENSG00000158941](https://www.ensembl.org/Homo_sapiens/geneview?gene=ENSG00000158941) |
| EAPP | E2F Associated Phosphoprotein | [ENSG00000129518](https://www.ensembl.org/Homo_sapiens/geneview?gene=ENSG00000129518) |
| FOXK2 | Forkhead Box K2 | [ENSG00000141568](https://www.ensembl.org/Homo_sapiens/geneview?gene=ENSG00000141568) |
| INCENP | Inner Centromere Protein | [ENSG00000149503](https://www.ensembl.org/Homo_sapiens/geneview?gene=ENSG00000149503) |
| MCM2 | Minichromosome Maintenance Complex Component 2 | [ENSG00000073111](https://www.ensembl.org/Homo_sapiens/geneview?gene=ENSG00000073111) |
| MSH6 | MutS Homolog 6 | [ENSG00000116062](https://www.ensembl.org/Homo_sapiens/geneview?gene=ENSG00000116062) |
| NUDT1 | Nudix Hydrolase 1 | [ENSG00000106268](https://www.ensembl.org/Homo_sapiens/geneview?gene=ENSG00000106268) |
| TP73 | Tumor Protein P73 | [ENSG00000078900](https://www.ensembl.org/Homo_sapiens/geneview?gene=ENSG00000078900) |
| PLK3 | Polo Like Kinase 3 | [ENSG00000173846](https://www.ensembl.org/Homo_sapiens/geneview?gene=ENSG00000173846) |
| PRIM2 | DNA Primase Subunit 2 | [ENSG00000146143](https://www.ensembl.org/Homo_sapiens/geneview?gene=ENSG00000146143) |
| RAD9A | RAD9 Checkpoint Clamp Component A | [ENSG00000172613](https://www.ensembl.org/Homo_sapiens/geneview?gene=ENSG00000172613) |
| MEX3C | Mex-3 RNA Binding Family Member C | [ENSG00000176624](https://www.ensembl.org/Homo_sapiens/geneview?gene=ENSG00000176624) |
| SMC1A | Structural Maintenance Of Chromosomes 1A | [ENSG00000072501](https://www.ensembl.org/Homo_sapiens/geneview?gene=ENSG00000072501) |
| TRIM28 | Tripartite Motif Containing 28 | [ENSG00000130726](https://www.ensembl.org/Homo_sapiens/geneview?gene=ENSG00000130726) |
| TRIM29 | Tripartite Motif Containing 29 | [ENSG00000137699](https://www.ensembl.org/Homo_sapiens/geneview?gene=ENSG00000137699) |
| USP2 | Ubiquitin Specific Peptidase 2 | [ENSG00000036672](https://www.ensembl.org/Homo_sapiens/geneview?gene=ENSG00000036672) |
| ERCC3 | ERCC Excision Repair 3, TFIIH Core Complex Helicase Subunit | [ENSG00000163161](https://www.ensembl.org/Homo_sapiens/geneview?gene=ENSG00000163161) |
| YWHAQ | Tyrosine 3-Monooxygenase/Tryptophan 5-Monooxygenase | [ENSG00000134308](https://www.ensembl.org/Homo_sapiens/geneview?gene=ENSG00000134308) |
| APEX1 | Apurinic/Apyrimidinic Endodeoxyribonuclease 1 | [ENSG00000100823](https://www.ensembl.org/Homo_sapiens/geneview?gene=ENSG00000100823) |
| BORA | BORA Aurora Kinase A Activator | [ENSG00000136122](https://www.ensembl.org/Homo_sapiens/geneview?gene=ENSG00000136122) |
| CAD | Carbamoyl-Phosphate Synthetase 2, Aspartate | [ENSG00000084774](https://www.ensembl.org/Homo_sapiens/geneview?gene=ENSG00000084774) |
| CDC23 | Cell Division Cycle 23 | [ENSG00000094880](https://www.ensembl.org/Homo_sapiens/geneview?gene=ENSG00000094880) |
| CDK1 | Cyclin Dependent Kinase 1 | [ENSG00000170312](https://www.ensembl.org/Homo_sapiens/geneview?gene=ENSG00000170312) |
| CDKN2C | Cyclin Dependent Kinase Inhibitor 2C | [ENSG00000123080](https://www.ensembl.org/Homo_sapiens/geneview?gene=ENSG00000123080) |
| CIP2A | Cellular Inhibitor Of PP2A | [ENSG00000163507](https://www.ensembl.org/Homo_sapiens/geneview?gene=ENSG00000163507) |
| DDB1 | Damage Specific DNA Binding Protein 1 | [ENSG00000167986](https://www.ensembl.org/Homo_sapiens/geneview?gene=ENSG00000167986) |
| MAPRE1 | Microtubule Associated Protein RP/EB Family Member 1 | [ENSG00000101367](https://www.ensembl.org/Homo_sapiens/geneview?gene=ENSG00000101367) |
| FOXM1 | Forkhead Box M1 | [ENSG00000111206](https://www.ensembl.org/Homo_sapiens/geneview?gene=ENSG00000111206) |
| JUNB | JunB Proto-Oncogene, AP-1 Transcription Factor Subunit | [ENSG00000171223](https://www.ensembl.org/Homo_sapiens/geneview?gene=ENSG00000171223) |
| MCM3 | Minichromosome Maintenance Complex Component 3 | [ENSG00000112118](https://www.ensembl.org/Homo_sapiens/geneview?gene=ENSG00000112118) |
| PWWP3A | PWWP Domain Containing 3A, DNA Repair Factor | [ENSG00000160953](https://www.ensembl.org/Homo_sapiens/geneview?gene=ENSG00000160953) |
| NUMA1 | Nuclear Mitotic Apparatus Protein 1 | [ENSG00000137497](https://www.ensembl.org/Homo_sapiens/geneview?gene=ENSG00000137497) |
| PARG | Poly(ADP-Ribose) Glycohydrolase | [ENSG00000227345](https://www.ensembl.org/Homo_sapiens/geneview?gene=ENSG00000227345) |
| PNKP | Polynucleotide Kinase 3'-Phosphatase | [ENSG00000039650](https://www.ensembl.org/Homo_sapiens/geneview?gene=ENSG00000039650) |
| PRIM1 | DNA Primase Subunit 1 | [ENSG00000198056](https://www.ensembl.org/Homo_sapiens/geneview?gene=ENSG00000198056) |
| UIMC1 | Ubiquitin Interaction Motif Containing 1 | [ENSG00000087206](https://www.ensembl.org/Homo_sapiens/geneview?gene=ENSG00000087206) |
| RNF2 | Ring Finger Protein 2 | [ENSG00000121481](https://www.ensembl.org/Homo_sapiens/geneview?gene=ENSG00000121481) |
| SMC2 | Structural Maintenance Of Chromosomes 2 | [ENSG00000136824](https://www.ensembl.org/Homo_sapiens/geneview?gene=ENSG00000136824) |
| TIGAR | TP53 Induced Glycolysis Regulatory Phosphatase | [ENSG00000078237](https://www.ensembl.org/Homo_sapiens/geneview?gene=ENSG00000078237) |
| TRRAP | Transformation/Transcription Domain Associated Protein | [ENSG00000196367](https://www.ensembl.org/Homo_sapiens/geneview?gene=ENSG00000196367) |
| USP4 | Ubiquitin Specific Peptidase 4 | [ENSG00000114316](https://www.ensembl.org/Homo_sapiens/geneview?gene=ENSG00000114316) |
| XPC | XPC Complex Subunit, DNA Damage Recognition And Repair Factor | [ENSG00000154767](https://www.ensembl.org/Homo_sapiens/geneview?gene=ENSG00000154767) |
| YWHAZ | Tyrosine 3-Monooxygenase/Tryptophan 5-Monooxygenase Activation Protein Theta | [ENSG00000134308](https://www.ensembl.org/Homo_sapiens/geneview?gene=ENSG00000134308) |
| ATRAID | All-Trans Retinoic Acid Induced Differentiation Factor | [ENSG00000138085](https://www.ensembl.org/Homo_sapiens/geneview?gene=ENSG00000138085) |
| BRCA1 | BRCA1 DNA Repair Associated | [ENSG00000012048](https://www.ensembl.org/Homo_sapiens/geneview?gene=ENSG00000012048) |
| CCNA2 | Cyclin A2 | [ENSG00000145386](https://www.ensembl.org/Homo_sapiens/geneview?gene=ENSG00000145386) |
| CDC25A | Cell Division Cycle 25A | [ENSG00000164045](https://www.ensembl.org/Homo_sapiens/geneview?gene=ENSG00000164045) |
| CDK10 | Cyclin Dependent Kinase 10 | [ENSG00000185324](https://www.ensembl.org/Homo_sapiens/geneview?gene=ENSG00000185324) |
| CDT1 | Chromatin Licensing And DNA Replication Factor 1 | [ENSG00000167513](https://www.ensembl.org/Homo_sapiens/geneview?gene=ENSG00000167513) |
| CIRBP | Cold Inducible RNA Binding Protein | [ENSG00000099622](https://www.ensembl.org/Homo_sapiens/geneview?gene=ENSG00000099622) |
| DDB2 | Damage Specific DNA Binding Protein 2 | [ENSG00000134574](https://www.ensembl.org/Homo_sapiens/geneview?gene=ENSG00000134574) |
| ENSA | Endosulfine Alpha | [ENSG00000143420](https://www.ensembl.org/Homo_sapiens/geneview?gene=ENSG00000143420) |
| GADD45A | Growth Arrest And DNA Damage Inducible Alpha | [ENSG00000116717](https://www.ensembl.org/Homo_sapiens/geneview?gene=ENSG00000116717) |
| MKI67 | Marker Of Proliferation Ki-67 | [ENSG00000148773](https://www.ensembl.org/Homo_sapiens/geneview?gene=ENSG00000148773) |
| MCM4 | Minichromosome Maintenance Complex Component 4 | [ENSG00000104738](https://www.ensembl.org/Homo_sapiens/geneview?gene=ENSG00000104738) |
| MUTYH | MutY DNA Glycosylase | [ENSG00000132781](https://www.ensembl.org/Homo_sapiens/geneview?gene=ENSG00000132781) |
| ORC1 | Origin Recognition Complex Subunit 1 | [ENSG00000085840](https://www.ensembl.org/Homo_sapiens/geneview?gene=ENSG00000085840) |
| PAXX | PAXX Non-Homologous End Joining Factor | [ENSG00000148362](https://www.ensembl.org/Homo_sapiens/geneview?gene=ENSG00000148362) |
| POLG | DNA Polymerase Gamma, Catalytic Subunit | [ENSG00000140521](https://www.ensembl.org/Homo_sapiens/geneview?gene=ENSG00000140521) |
| PTPN12 | Protein Tyrosine Phosphatase Non-Receptor Type 12 | [ENSG00000127947](https://www.ensembl.org/Homo_sapiens/geneview?gene=ENSG00000127947) |
| RB1 | RB Transcriptional Corepressor 1 | [ENSG00000139687](https://www.ensembl.org/Homo_sapiens/geneview?gene=ENSG00000139687) |
| RPA1 | Replication Protein A1 | [ENSG00000132383](https://www.ensembl.org/Homo_sapiens/geneview?gene=ENSG00000132383) |
| SMC3 | Structural Maintenance Of Chromosomes 3 | [ENSG00000108055](https://www.ensembl.org/Homo_sapiens/geneview?gene=ENSG00000108055) |
| KAT5 | Lysine Acetyltransferase 5 | [ENSG00000172977](https://www.ensembl.org/Homo_sapiens/geneview?gene=ENSG00000172977) |
| TTK | TTK Protein Kinase | [ENSG00000112742](https://www.ensembl.org/Homo_sapiens/geneview?gene=ENSG00000112742) |
| UVRAG | UV Radiation Resistance Associated | [ENSG00000198382](https://www.ensembl.org/Homo_sapiens/geneview?gene=ENSG00000198382) |
| XRCC1 | X-Ray Repair Cross Complementing 1 | [ENSG00000073050](https://www.ensembl.org/Homo_sapiens/geneview?gene=ENSG00000073050) |
| TP53BP1 | Tumor Protein P53 Binding Protein 1 | [ENSG00000067369](https://www.ensembl.org/Homo_sapiens/geneview?gene=ENSG00000067369) |
| APTX | Aprataxin | [ENSG00000137074](https://www.ensembl.org/Homo_sapiens/geneview?gene=ENSG00000137074) |
| BRCA2 | BRCA2 DNA Repair Associated | [ENSG00000139618](https://www.ensembl.org/Homo_sapiens/geneview?gene=ENSG00000139618) |
| CCNB1 | Cyclin B1 | [ENSG00000134057](https://www.ensembl.org/Homo_sapiens/geneview?gene=ENSG00000134057) |
| CDC25B | Cell Division Cycle 25B | [ENSG00000101224](https://www.ensembl.org/Homo_sapiens/geneview?gene=ENSG00000101224) |
| CDK11B | Cyclin Dependent Kinase 11B | [ENSG00000248333](https://www.ensembl.org/Homo_sapiens/geneview?gene=ENSG00000248333) |
| CEND1 | Cell Cycle Exit And Neuronal Differentiation 1 | [ENSG00000184524](https://www.ensembl.org/Homo_sapiens/geneview?gene=ENSG00000184524) |
| CSNK1A1 | Casein Kinase 1 Alpha 1 | [ENSG00000113712](https://www.ensembl.org/Homo_sapiens/geneview?gene=ENSG00000113712) |
| DDIT4 | DNA Damage Inducible Transcript 4 | [ENSG00000168209](https://www.ensembl.org/Homo_sapiens/geneview?gene=ENSG00000168209) |
| ERCC1 | ERCC Excision Repair 1, Endonuclease Non-Catalytic Subunit | [ENSG00000012061](https://www.ensembl.org/Homo_sapiens/geneview?gene=ENSG00000012061) |
| GMNN | Geminin DNA Replication Inhibitor | ENSG00000112312 |
| KIF11 | Kinesin Family Member 11 | [ENSG00000138160](https://www.ensembl.org/Homo_sapiens/geneview?gene=ENSG00000138160) |
| MCM7 | Minichromosome Maintenance Complex Component 7 | [ENSG00000166508](https://www.ensembl.org/Homo_sapiens/geneview?gene=ENSG00000166508) |
| PKMYT1 | Protein Kinase, Membrane Associated Tyrosine/Threonine 1 | [ENSG00000127564](https://www.ensembl.org/Homo_sapiens/geneview?gene=ENSG00000127564) |
| ORC2 | Origin Recognition Complex Subunit 2 | [ENSG00000115942](https://www.ensembl.org/Homo_sapiens/geneview?gene=ENSG00000115942) |
| PBK | PDZ Binding Kinase | [ENSG00000168078](https://www.ensembl.org/Homo_sapiens/geneview?gene=ENSG00000168078) |
| POLH | DNA Polymerase Eta | [ENSG00000170734](https://www.ensembl.org/Homo_sapiens/geneview?gene=ENSG00000170734) |
| PTPRA | Protein Tyrosine Phosphatase Receptor Type A | [ENSG00000132670](https://www.ensembl.org/Homo_sapiens/geneview?gene=ENSG00000132670) |
| RBL2 | RB Transcriptional Corepressor Like 2 | ENSG00000103479 |
| RRM1 | Ribonucleotide Reductase Catalytic Subunit M1 | [ENSG00000167325](https://www.ensembl.org/Homo_sapiens/geneview?gene=ENSG00000167325) |
| SMC4 | Structural Maintenance Of Chromosomes 4 | [ENSG00000113810](https://www.ensembl.org/Homo_sapiens/geneview?gene=ENSG00000113810) |
| TLK1 | Tousled Like Kinase 1 | [ENSG00000198586](https://www.ensembl.org/Homo_sapiens/geneview?gene=ENSG00000198586) |
| TYMS | Thymidylate Synthetase | [ENSG00000176890](https://www.ensembl.org/Homo_sapiens/geneview?gene=ENSG00000176890) |
| VCP | Valosin Containing Protein | [ENSG00000165280](https://www.ensembl.org/Homo_sapiens/geneview?gene=ENSG00000165280) |
| YY1 | YY1 Transcription Factor | [ENSG00000100811](https://www.ensembl.org/Homo_sapiens/geneview?gene=ENSG00000100811) |
| ACD | ACD Shelterin Complex Subunit And Telomerase Recruitment Factor | [ENSG00000102977](https://www.ensembl.org/Homo_sapiens/geneview?gene=ENSG00000102977) |
| ARPP19 | CAMP Regulated Phosphoprotein 19 | [ENSG00000128989](https://www.ensembl.org/Homo_sapiens/geneview?gene=ENSG00000128989) |
| BRCC3 | BRCA1/BRCA2-Containing Complex Subunit 3 | [ENSG00000185515](https://www.ensembl.org/Homo_sapiens/geneview?gene=ENSG00000185515) |
| CCND1 | Cyclin D1 | [ENSG00000110092](https://www.ensembl.org/Homo_sapiens/geneview?gene=ENSG00000110092) |
| CDC25C | Cell Division Cycle 25C | [ENSG00000158402](https://www.ensembl.org/Homo_sapiens/geneview?gene=ENSG00000158402) |
| CDK12 | Cyclin Dependent Kinase 12 | [ENSG00000167258](https://www.ensembl.org/Homo_sapiens/geneview?gene=ENSG00000167258) |
| CENPA | Centromere Protein A | [ENSG00000115163](https://www.ensembl.org/Homo_sapiens/geneview?gene=ENSG00000115163) |
| CSNK2A1 | Casein Kinase 2 Alpha 1 | [ENSG00000101266](https://www.ensembl.org/Homo_sapiens/geneview?gene=ENSG00000101266) |
| DDX3X | DEAD-Box Helicase 3 X-Linked | [ENSG00000215301](https://www.ensembl.org/Homo_sapiens/geneview?gene=ENSG00000215301) |
| ERCC2 | ERCC Excision Repair 2, TFIIH Core Complex Helicase Subunit | [ENSG00000104884](https://www.ensembl.org/Homo_sapiens/geneview?gene=ENSG00000104884) |
| GSTP1 | Glutathione S-Transferase Pi 1 | [ENSG00000084207](https://www.ensembl.org/Homo_sapiens/geneview?gene=ENSG00000084207) |
| XRCC6 | X-Ray Repair Cross Complementing 6 | [ENSG00000196419](https://www.ensembl.org/Homo_sapiens/geneview?gene=ENSG00000196419) |
| MCPH1 | Microcephalin 1 | [ENSG00000147316](https://www.ensembl.org/Homo_sapiens/geneview?gene=ENSG00000147316) |
| NAA10 | N-Alpha-Acetyltransferase 10, NatA Catalytic Subunit | [ENSG00000102030](https://www.ensembl.org/Homo_sapiens/geneview?gene=ENSG00000102030) |
| ORC6 | Origin Recognition Complex Subunit 6 | [ENSG00000091651](https://www.ensembl.org/Homo_sapiens/geneview?gene=ENSG00000091651) |
| PCM1 | Pericentriolar Material 1 | [ENSG00000078674](https://www.ensembl.org/Homo_sapiens/geneview?gene=ENSG00000078674) |
| POLR2A | RNA Polymerase II Subunit A | [ENSG00000181222](https://www.ensembl.org/Homo_sapiens/geneview?gene=ENSG00000181222) |
| RAD17 | RAD17 Checkpoint Clamp Loader Component | [ENSG00000152942](https://www.ensembl.org/Homo_sapiens/geneview?gene=ENSG00000152942) |
| RBMX | RNA Binding Motif Protein X-Linked | [ENSG00000147274](https://www.ensembl.org/Homo_sapiens/geneview?gene=ENSG00000147274) |
| S100A6 | S100 Calcium Binding Protein A6 | [ENSG00000197956](https://www.ensembl.org/Homo_sapiens/geneview?gene=ENSG00000197956) |
| SMG1 | SMG1 Nonsense Mediated MRNA Decay Associated PI3K Related Kinase | [ENSG00000157106](https://www.ensembl.org/Homo_sapiens/geneview?gene=ENSG00000157106) |
| TMPO | Thymopoietin | [ENSG00000120802](https://www.ensembl.org/Homo_sapiens/geneview?gene=ENSG00000120802) |
| UBE2A | Ubiquitin Conjugating Enzyme E2 A | ENSG00000077721 |
| VIM | Vimentin | [ENSG00000026025](https://www.ensembl.org/Homo_sapiens/geneview?gene=ENSG00000026025) |
| ADD1 | Adducin 1 | [ENSG00000087274](https://www.ensembl.org/Homo_sapiens/geneview?gene=ENSG00000087274) |
| DCLRE1C | DNA Cross-Link Repair 1C | [ENSG00000152457](https://www.ensembl.org/Homo_sapiens/geneview?gene=ENSG00000152457) |
| BRD2 | Bromodomain Containing 2 | [ENSG00000204256](https://www.ensembl.org/Homo_sapiens/geneview?gene=ENSG00000204256) |
| CCND2 | Cyclin D2 | [ENSG00000118971](https://www.ensembl.org/Homo_sapiens/geneview?gene=ENSG00000118971) |
| CDC27 | Cell Division Cycle 27 | [ENSG00000004897](https://www.ensembl.org/Homo_sapiens/geneview?gene=ENSG00000004897) |
| CDK16 | Cyclin Dependent Kinase 16 | [ENSG00000102225](https://www.ensembl.org/Homo_sapiens/geneview?gene=ENSG00000102225) |
| CENPE | Centromere Protein E | [ENSG00000138778](https://www.ensembl.org/Homo_sapiens/geneview?gene=ENSG00000138778) |
| CKAP5 | Cytoskeleton Associated Protein 5 | [ENSG00000175216](https://www.ensembl.org/Homo_sapiens/geneview?gene=ENSG00000175216) |
| DMAP1 | DNA Methyltransferase 1 Associated Protein 1 | [ENSG00000178028](https://www.ensembl.org/Homo_sapiens/geneview?gene=ENSG00000178028) |
| ERCC4 | ERCC Excision Repair 4, Endonuclease Catalytic Subunit | [ENSG00000175595](https://www.ensembl.org/Homo_sapiens/geneview?gene=ENSG00000175595) |
| H2AX | H2A.X Variant Histone | [ENSG00000188486](https://www.ensembl.org/Homo_sapiens/geneview?gene=ENSG00000188486) |
| XRCC5 | X-Ray Repair Cross Complementing 5 | [ENSG00000079246](https://www.ensembl.org/Homo_sapiens/geneview?gene=ENSG00000079246) |
| MDM2 | MDM2 Proto-Oncogene | [ENSG00000135679](https://www.ensembl.org/Homo_sapiens/geneview?gene=ENSG00000135679) |
| BABAM1 | BRISC And BRCA1 A Complex Member 1 | [ENSG00000105393](https://www.ensembl.org/Homo_sapiens/geneview?gene=ENSG00000105393) |
| OTUB1 | OTU Deubiquitinase, Ubiquitin Aldehyde Binding 1 | [ENSG00000167770](https://www.ensembl.org/Homo_sapiens/geneview?gene=ENSG00000167770) |
| PCNA | Proliferating Cell Nuclear Antigen | [ENSG00000132646](https://www.ensembl.org/Homo_sapiens/geneview?gene=ENSG00000132646) |
| PPM1D | Protein Phosphatase, Mg2+/Mn2+ Dependent 1D | [ENSG00000170836](https://www.ensembl.org/Homo_sapiens/geneview?gene=ENSG00000170836) |
| RAD18 | RAD18 E3 Ubiquitin Protein Ligase | [ENSG00000070950](https://www.ensembl.org/Homo_sapiens/geneview?gene=ENSG00000070950) |
| RCC1 | Regulator Of Chromosome Condensation 1 | [ENSG00000180198](https://www.ensembl.org/Homo_sapiens/geneview?gene=ENSG00000180198) |
| PTTG1 | PTTG1 Regulator Of Sister Chromatid Separation, Securin | [ENSG00000164611](https://www.ensembl.org/Homo_sapiens/geneview?gene=ENSG00000164611) |
| SPART | Spartin | [ENSG00000133104](https://www.ensembl.org/Homo_sapiens/geneview?gene=ENSG00000133104) |
| TOP1 | DNA Topoisomerase I | [ENSG00000198900](https://www.ensembl.org/Homo_sapiens/geneview?gene=ENSG00000198900) |
| UBE2B | Ubiquitin Conjugating Enzyme E2 B | [ENSG00000119048](https://www.ensembl.org/Homo_sapiens/geneview?gene=ENSG00000119048) |
| VRK1 | VRK Serine/Threonine Kinase 1 | [ENSG00000100749](https://www.ensembl.org/Homo_sapiens/geneview?gene=ENSG00000100749) |
| ALKBH7 | AlkB Homolog 7 | [ENSG00000125652](https://www.ensembl.org/Homo_sapiens/geneview?gene=ENSG00000125652) |
| ATM | ATM Serine/Threonine Kinase | [ENSG00000149311](https://www.ensembl.org/Homo_sapiens/geneview?gene=ENSG00000149311) |
| BABAM2 | BRISC And BRCA1 A Complex Member 2 | [ENSG00000158019](https://www.ensembl.org/Homo_sapiens/geneview?gene=ENSG00000158019) |
| CCND3 | Cyclin D3 | [ENSG00000112576](https://www.ensembl.org/Homo_sapiens/geneview?gene=ENSG00000112576) |
| CDC37 | Cell Division Cycle 37, HSP90 Cochaperone | [ENSG00000105401](https://www.ensembl.org/Homo_sapiens/geneview?gene=ENSG00000105401) |
| CDK2 | Cyclin Dependent Kinase 2 | [ENSG00000123374](https://www.ensembl.org/Homo_sapiens/geneview?gene=ENSG00000123374) |
| CENPF | Centromere Protein F | [ENSG00000117724](https://www.ensembl.org/Homo_sapiens/geneview?gene=ENSG00000117724) |
| CLASP2 | Cytoplasmic Linker Associated Protein 2 | [ENSG00000163539](https://www.ensembl.org/Homo_sapiens/geneview?gene=ENSG00000163539) |
| PRKDC | Protein Kinase, DNA-Activated, Catalytic Subunit | [ENSG00000253729](https://www.ensembl.org/Homo_sapiens/geneview?gene=ENSG00000253729) |
| FANCA | FA Complementation Group A | [ENSG00000187741](https://www.ensembl.org/Homo_sapiens/geneview?gene=ENSG00000187741) |
| H3C1 | H3 Clustered Histone 1 | [ENSG00000275714](https://www.ensembl.org/Homo_sapiens/geneview?gene=ENSG00000275714) |
| LMNA | Lamin A/C | [ENSG00000160789](https://www.ensembl.org/Homo_sapiens/geneview?gene=ENSG00000160789) |
| NTMT1 | N-Terminal Xaa-Pro-Lys N-Methyltransferase 1 | [ENSG00000148335](https://www.ensembl.org/Homo_sapiens/geneview?gene=ENSG00000148335) |
| NBN | Nibrin | [ENSG00000104320](https://www.ensembl.org/Homo_sapiens/geneview?gene=ENSG00000104320) |
| CDKN2A | Cyclin Dependent Kinase Inhibitor 2A | [ENSG00000147889](https://www.ensembl.org/Homo_sapiens/geneview?gene=ENSG00000147889) |
| PHB1 | Prohibitin 1 | [ENSG00000167085](https://www.ensembl.org/Homo_sapiens/geneview?gene=ENSG00000167085) |
| PPP1CA | Protein Phosphatase 1 Catalytic Subunit Alpha | [ENSG00000172531](https://www.ensembl.org/Homo_sapiens/geneview?gene=ENSG00000172531) |
| RAD21 | RAD21 Cohesin Complex Component | [ENSG00000164754](https://www.ensembl.org/Homo_sapiens/geneview?gene=ENSG00000164754) |
| RCHY1 | Ring Finger And CHY Zinc Finger Domain Containing 1 | [ENSG00000163743](https://www.ensembl.org/Homo_sapiens/geneview?gene=ENSG00000163743) |
| SESN2 | Sestrin 2 | [ENSG00000130766](https://www.ensembl.org/Homo_sapiens/geneview?gene=ENSG00000130766) |
| STAG2 | Stromal Antigen 2 | [ENSG00000101972](https://www.ensembl.org/Homo_sapiens/geneview?gene=ENSG00000101972) |
| TOP2A | DNA Topoisomerase II Alpha | [ENSG00000131747](https://www.ensembl.org/Homo_sapiens/geneview?gene=ENSG00000131747) |
| UBE2C | Ubiquitin Conjugating Enzyme E2 C | [ENSG00000175063](https://www.ensembl.org/Homo_sapiens/geneview?gene=ENSG00000175063) |
| VRK3 | VRK Serine/Threonine Kinase 3 | [ENSG00000105053](https://www.ensembl.org/Homo_sapiens/geneview?gene=ENSG00000105053) |
| ANP32A | Acidic Nuclear Phosphoprotein 32 Family Member A | [ENSG00000140350](https://www.ensembl.org/Homo_sapiens/geneview?gene=ENSG00000140350) |
| ATR | ATR Serine/Threonine Kinase | [ENSG00000175054](https://www.ensembl.org/Homo_sapiens/geneview?gene=ENSG00000175054) |
| BRIP1 | BRCA1 Interacting Helicase 1 | [ENSG00000136492](https://www.ensembl.org/Homo_sapiens/geneview?gene=ENSG00000136492) |
| CCNE1 | Cyclin E1 | [ENSG00000105173](https://www.ensembl.org/Homo_sapiens/geneview?gene=ENSG00000105173) |
| CDC45 | Cell Division Cycle 45 | [ENSG00000093009](https://www.ensembl.org/Homo_sapiens/geneview?gene=ENSG00000093009) |
| CDK4 | Cyclin Dependent Kinase 4 | [ENSG00000135446](https://www.ensembl.org/Homo_sapiens/geneview?gene=ENSG00000135446) |
| CENPI | Centromere Protein I | [ENSG00000102384](https://www.ensembl.org/Homo_sapiens/geneview?gene=ENSG00000102384) |
| CLSPN | Claspin | [ENSG00000092853](https://www.ensembl.org/Homo_sapiens/geneview?gene=ENSG00000092853) |
| DPYD | Dihydropyrimidine Dehydrogenase | [ENSG00000188641](https://www.ensembl.org/Homo_sapiens/geneview?gene=ENSG00000188641) |
| FANCB | FA Complementation Group B | [ENSG00000181544](https://www.ensembl.org/Homo_sapiens/geneview?gene=ENSG00000181544) |
| HCFC1 | Host Cell Factor C1 | [ENSG00000172534](https://www.ensembl.org/Homo_sapiens/geneview?gene=ENSG00000172534) |
| LATS1 | Large Tumor Suppressor Kinase 1 | [ENSG00000131023](https://www.ensembl.org/Homo_sapiens/geneview?gene=ENSG00000131023) |
| MGMT | O-6-Methylguanine-DNA Methyltransferase | [ENSG00000170430](https://www.ensembl.org/Homo_sapiens/geneview?gene=ENSG00000170430) |
| NCAPD3 | Non-SMC Condensin II Complex Subunit D3 | ENSG00000151503 |
| CDKN1A | Cyclin Dependent Kinase Inhibitor 1A | [ENSG00000124762](https://www.ensembl.org/Homo_sapiens/geneview?gene=ENSG00000124762) |
| PHLDA3 | Pleckstrin Homology Like Domain Family A Member 3 | [ENSG00000174307](https://www.ensembl.org/Homo_sapiens/geneview?gene=ENSG00000174307) |
| PPP2CA | Protein Phosphatase 2 Catalytic Subunit Alpha | [ENSG00000113575](https://www.ensembl.org/Homo_sapiens/geneview?gene=ENSG00000113575) |
| RAD23A | RAD23 Homolog A, Nucleotide Excision Repair Protein | [ENSG00000179262](https://www.ensembl.org/Homo_sapiens/geneview?gene=ENSG00000179262) |
| RECQL | RecQ Like Helicase | [ENSG00000004700](https://www.ensembl.org/Homo_sapiens/geneview?gene=ENSG00000004700) |
| SKP2 | S-Phase Kinase Associated Protein 2 | [ENSG00000145604](https://www.ensembl.org/Homo_sapiens/geneview?gene=ENSG00000145604) |
| TACC3 | Transforming Acidic Coiled-Coil Containing Protein 3 | [ENSG00000013810](https://www.ensembl.org/Homo_sapiens/geneview?gene=ENSG00000013810) |
| TOPBP1 | DNA Topoisomerase II Binding Protein 1 | [ENSG00000163781](https://www.ensembl.org/Homo_sapiens/geneview?gene=ENSG00000163781) |
| UBE2N | Ubiquitin Conjugating Enzyme E2 N | [ENSG00000177889](https://www.ensembl.org/Homo_sapiens/geneview?gene=ENSG00000177889) |
| WAPL | WAPL Cohesin Release Factor | [ENSG00000062650](https://www.ensembl.org/Homo_sapiens/geneview?gene=ENSG00000062650) |

**Additional file 1: Table S2** Details of 27 immune checkpoints.

| **Gene symbol** | **Full name** | **Ensemble ID** |
| --- | --- | --- |
| IDO1 | indoleamine 2,3-dioxygenase 1 | ENSG00000131203 |
| CTLA4 | cytotoxic T-lymphocyte associated protein 4 | ENSG00000163599 |
| TNFRSF9 | TNF receptor superfamily member 9 | ENSG00000049249 |
| ICOS | inducible T cell costimulator | ENSG00000163600 |
| CD80 | CD80 molecule | ENSG00000121594 |
| TIGIT | T cell immunoreceptor with Ig and ITIM domains | ENSG00000181847 |
| TNFSF9 | TNF superfamily member 9 | ENSG00000125657 |
| CD86 | CD86 molecule | ENSG00000114013 |
| PDCD1 | prephenate dehydratase 1 | ENSG00000188389 |
| LAIR1 | leukocyte associated immunoglobulin like receptor 1 | ENSG00000167613 |
| TNFSF15 | TNF superfamily member 15 | ENSG00000181634 |
| TNFRSF14 | TNF receptor superfamily member 14 | ENSG00000157873 |
| CD276 | CD276 molecule | ENSG00000103855 |
| TNFRSF4 | TNF receptor superfamily member 4 | ENSG00000186827 |
| CD274 | CD274 molecule | ENSG00000120217 |
| HAVCR2 | hepatitis A virus cellular receptor 2 | ENSG00000135077 |
| CD27 | CD27 molecule | ENSG00000139193 |
| LGALS9 | galectin 9 | ENSG00000168961 |
| CD28 | CD28 molecule | ENSG00000178562 |
| TNFRSF25 | TNF receptor superfamily member 25 | ENSG00000215788 |
| VTCN1 | V-set domain containing T cell activation inhibitor 1 | ENSG00000134258 |
| CD44 | CD44 Molecule | ENSG00000026508 |
| TNFRSF18 | TNF Receptor Superfamily Member 18 | ENSG00000186891 |
| CD200R1 | CD200 Receptor 1 | ENSG00000163606 |
| TNFSF4 | TNF Superfamily Member 4 | ENSG00000117586 |
| CD200 | CD200 Molecule | ENSG00000091972 |
| NRP1 | Neuropilin 1 | ENSG00000099250 |

**Additional file 1: Table S3** Details of gene sets of DNA damage repair, X-ray, and UV response for ssGSEA analysis.

| **Standard name** | **Genes** |
| --- | --- |
| Base excision repair (BER) | UNG, SMUG1, MBD4, TDG, OGG1, MUTYH (MYH), NTHL1 (NTH1), MPG, NEIL1, NEIL2, NEIL3 |
| Chromatin Structure and Modification | H2AX, CHAF1A, SETMAR, ATRX |
| Direct reversal of damage | MGMT, ABH2, DEPC1 |
| DNA polymerases | POLA1, POLB, POLD1, POLD2, POLD3, POLD4, POLE, POLE2, POLE3, POLE4, REV3L, MAD2L2, REV1, POLG, POLH, POLI, POLQ, POLK, POLL, POLM, POLN, PRIMPOL, DNTT |
| Editing and processing nucleases | FEN1, FAN1, TREX1, TREX2, EXO1, APTX, SPO11, ENDOV, DNA2, DCLRE1A, DCLRE1B, EXO5 |
| Fanconi anemia | FANCA, FANCB, FANCC, BRCA2, FANCD2, FANCE, FANCF, FANCG, FANCI, BRIP1, FANCL, FANCM, PALB2, RAD51C, SLX4, FAAP20, FAAP24, FAAP100, UBE2T |
| Homologous recombination (HR) | RAD51, RAD51B, RAD51D, HELQ, SWI5, SWSAP1, ZSWIM7, SPIDR, PDS5B, DMC1, XRCC2, XRCC3, RAD52, RAD54L, RAD54B, BRCA1, BARD1, ABRAXAS1, PAXIP1, SMC5, SMC6, SHLD1, SHLD2, SHLD3, SEM1, RAD50, MRE11A, NBN, RBBP8, MUS81, EME1, EME2, SLX1A, SLX1B, GEN1 |
| Mismatch excision repair (MMR) | MSH2, MSH3, MSH6, MLH1, PMS2, MSH4, MSH5, MLH3, PMS1, PMS2P3, HFM1 |
| Modulation of nucleotide pools | NUDT1, DUT, RRM2B, PARK7, DNPH1, NUDT15, NUDT18 |
| Nucleotide excision repair (NER) | XPC, RAD23B, CETN2, RAD23A, XPA, DDB1, DDB2, RPA1, RPA2, RPA3, TFIIH, ERCC3, ERCC2, GTF2H1, GTF2H2, GTF2H3, GTF2H4, GTF2H5, GTF2E2, CDK7, CCNH, MNAT1, ERCC5, ERCC1, ERCC4, LIG1 |
| Non-homologous end-joining (NHEJ) | XRCC6, XRCC5, PRKDC, LIG4, XRCC4, DCLRE1C, NHEJ1 |
| Other conserved DNA damage response genes | ATR, ATRIP, MDC1, PCNA, RAD1, RAD9A, HUS1, RAD17, CHEK1, CHEK2, TP53, TP53BP1, RIF1, TOPBP1, CLK2, PER1 |
| Poly(ADP-ribose) polymerase (PARP) enzymes that bind to DNA | ADPRT, ADPRTL2, ADPRTL3, PARG, PARPBP |
| Repair of DNA-protein crosslinks | TDP1, TDP2, SPRTN |
| Ubiquitination and modification | UBE2A, UBE2B, RAD18, SHPRH, HLTF, RNF168, RNF8, RNF4, UBE2V2, UBE2N, USP1, WDR48, HERC2 |
| GOBP_RESPONSE_TO_X_RAY | ATM, DNM2, GATA3, HAMP, HMGA2, NIPBL, NUCKS1, PRAP1, SFRP1, SFRP2, TP53BP1, XRCC5, XRCC6 |
| GOBP_RESPONSE_TO_UV | ACTR5, AKT1, AQP1, ATF4, ATR, AURKB, BAK1, BAX, BCL2, BCL3, BMF, BRCA2, BRSK1, C9orf116, CARD16, CASP3, CASP7, CASP9, CAT, CCAR2, CCND1, CDC25A, CDKN1A, CDKN2D, CERS1, CHEK1, CIRBP, COPS9, CREBBP, CRIP1, CUL4B, DCUN1D3, DDB1, DDB2, DHX36, DTL, EGFR, EIF2AK4, EIF2S1, ELANE, EP300, ERCC1, ERCC2, ERCC3, ERCC4, ERCC5, ERCC6, ERCC8, FEN1, FMR1, GPX1, GTF2H2, H2AW, HUS1, HYAL1, HYAL2, HYAL3, IL12A, IL12B, IMPACT, INO80, IVL, KDM1A, MAP2K7, MAP3K4, MAP4K3, MAPK13, MAPK8, MC1R, MEN1, METTL3, MFAP4, MME, MMP1, MMP2, MMP3, MMP9, MSH2, MSH6, MYC, N4BP1, NEDD4, NFATC4, NOC2L, NPM1, NSMCE3, OPN1SW, OPN3, OPN5, PARP1, PBK, PCLAF, PCNA, PIK3R1, PML, POLA1, POLD1, POLD3, POLH, POLK, PPID, PRIMPOL, PRKAA1, PRKCD, PTGS2, PTPRK, RELA, REV1, RHBDD1, RHNO1, RO60, RPL26, RUVBL2, SCARA3, SDE2, SDF4, SERPINB13, SIRT1, SMPD1, SPRTN, ST20, STK11, TAF1, TIMP1, TIPIN, TMEM161A, TP53, TP53I13, TP53INP1, TREX1, TRIAP1, TRIM32, TYR, UBE2A, UBE2B, UBE4B, USF1, USP1, USP28, USP47, UVSSA, WRN, XPA, XPC, YY1, ZBTB1, ZRANB3 |
| GOBP_RESPONSE_TO_UV_A | AKT1, CCND1, CERS1, EGFR, MME, MMP1, MMP2, MMP3, MMP9, OPN1SW, OPN3, OPN5, PPID, TIMP1 |
| GOBP_RESPONSE_TO_UV_B | BCL2, CDKN1A, CRIP1, ERCC6, HYAL1, HYAL2, HYAL3, IL12A, IL12B, IVL, MFAP4, MME, MSH2, RELA, STK11, XPC |
| GOBP_RESPONSE_TO_UV_C | BAK1, BCL3, BRCA2, C9orf116, CARD16, DCUN1D3, ERCC5, IMPACT, MAP3K4, POLH, ST20, TP53, WRN, YY1 |
| GOBP_RESPONSE_TO_X_RAY | ANXA1, ATM, BLM, BRCA2, BRCC3, CASP3, CCND1, DNM2, ERCC1, ERCC6, ERCC8, GATA3, HAMP, HMGA2, IKBIP, KARS1, LIG4, MSH2, NIPBL, NUCKS1, PRAP1, RAD51, SFRP1, SFRP2, THBD, TP53, TP53BP1, XRCC2, XRCC4, XRCC5, XRCC6, XRRA1 |
| GOBP_UV_DAMAGE_EXCISION_REPAIR | ACTR5, CUL4B, DDB1, DDB2, ERCC1, H2AW, INO80, MC1R, POLA1, POLD3, SIRT1, TREX1, XPA, XPC |

**Additional file 1: Table S4** The correlated small molecule drugs for two risk groups obtained from the CMap. ( Positive correlation, Score>0; Negative correlation, Score<0 )

| Rank | Score | ID | Name | Description |
| --- | --- | --- | --- | --- |
| 1 | 99.92 | BRD-K44442813 | pidotimod | Interferon receptor agonist |
| 2 | 99.91 | BRD-K80527266 | triacsin-c | Adrenergic receptor antagonist |
| 3 | 99.88 | BRD-K32536677 | AGK-2 | SIRT inhibitor |
| 4 | 99.86 | BRD-K55703048 | latrepirdine | Glutamate receptor antagonist |
| 5 | 99.85 | BRD-K41260949 | valproic-acid | HDAC inhibitor |
| 6 | 99.82 | BRD-K17349619 | HLI-373 | MDM inhibitor |
| 7 | 99.73 | BRD-A64228451 | terreic-acid | BTK inhibitor |
| 8 | 99.66 | BRD-K59184148 | SB-216763 | Glycogen synthase kinase inhibitor |
| 9 | 99.62 | BRD-U97083655 | teicoplanin | Bacterial cell wall synthesis inhibitor |
| 10 | 99.57 | BRD-K33572481 | taurodeoxycholic-acid | Bile acid |
| 11 | 99.41 | BRD-K32107296 | temozolomide | DNA alkylating agent |
| 12 | 99.28 | BRD-K77625572 | etomoxir | Carnitine palmitoyltransferase inhibitor |
| 13 | 99.28 | BRD-K06878038 | deferiprone | Chelating agent |
| 14 | 99.26 | BRD-K93918653 | quizartinib | FLT3 inhibitor |
| 15 | 99.21 | BRD-K14696368 | 9-methyl-5H-6-thia-4,5-diaza-chrysene-6,6-dioxide | NFkB pathway inhibitor |
| 7969 | -99.95 | BRD-K30097969 | pitavastatin | HMGCR inhibitor |
| 7968 | -99.84 | BRD-K18787491 | U-0126 | MEK inhibitor |
| 7967 | -99.61 | BRD-M86331534 | pyrvinium-pamoate | AKT inhibitor |
| 7966 | -99.59 | BRD-K12867552 | THM-I-94 | HDAC inhibitor |
| 7965 | -99.57 | BRD-K37798499 | etoposide | Topoisomerase inhibitor |
| 7964 | -99.56 | BRD-A35588707 | teniposide | Topoisomerase inhibitor |
| 7963 | -99.38 | BRD-K03109492 | NSC-663284 | CDC inhibitor |
| 7962 | -99.34 | BRD-K91370081 | anisomycin | DNA synthesis inhibitor |
| 7961 | -99.31 | BRD-A94756469 | digoxin | ATPase inhibitor |
| 7960 | -99.29 | BRD-K57080016 | selumetinib | MEK inhibitor |
| 7959 | -99.26 | BRD-K80348542 | cephaeline | Protein synthesis inhibitor |
| 7958 | -99.2 | BRD-K21680192 | mitoxantrone | Topoisomerase inhibitor |
| 7957 | -99.19 | BRD-A25687296 | emetine | Protein synthesis inhibitor |
| 7956 | -99.18 | BRD-K63606607 | bufalin | ATPase inhibitor |
| 7955 | -99.13 | BRD-K28907958 | CD-437 | Retinoid receptor agonist |
