# Supplementary material for: The DDR-related gene signature with cell cycle checkpoint function predicts prognosis, immune activity, and chemoradiotherapy response in lung adenocarcinoma
Source: Respir Res. 2022 Jul 15;23:190. doi: 10.1186/s12931-022-02110-w (PMC9288070; doi:10.1186/s12931-022-02110-w)
Supplement: Supplementary file 2 — Additional file 2. Additional tables. [file 12931_2022_2110_MOESM2_ESM.docx]

**
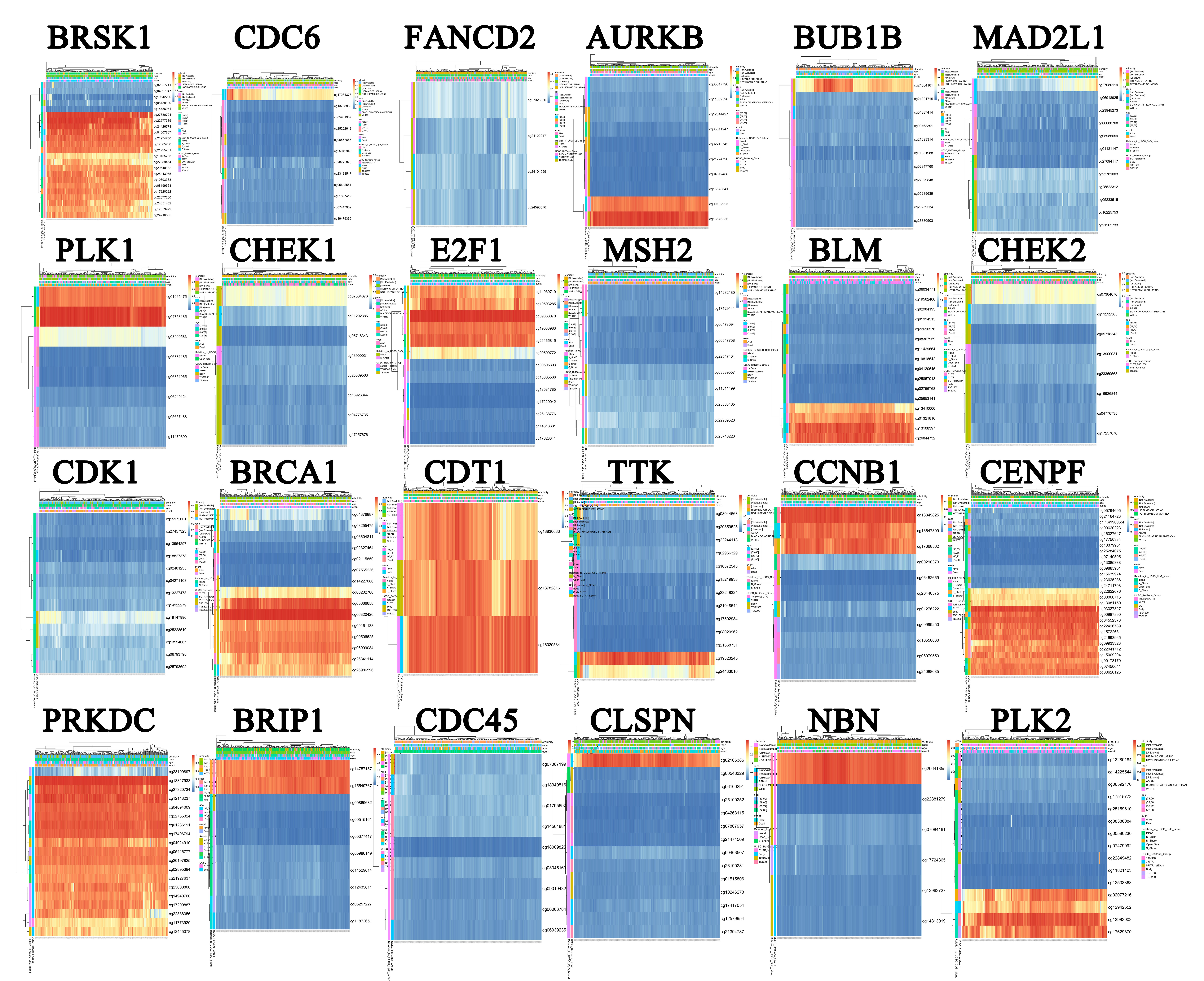
**

**Additional file 2: Fig. S1** The heatmap for methylation levels of 24 DCGs CpG island in LUAD (blue: low methylation level, orange: methylation levels).





**Additional file 2: Fig. S2** Prognosis model based on the DCGs signature. (**A**, **B**) The selection of optimal cutoff value for risk score by X-tile software. (**C**) Distribution of patients based on the risk score. (**D**, **E**) Analysis of the survival rate and survival status in the two risk groups. (**F**) Principal component analysis (PCA) of the 4-DCGs signature. (**G**) The time-dependent receiver operating characteristic (ROC) of 4-DCGs signature.


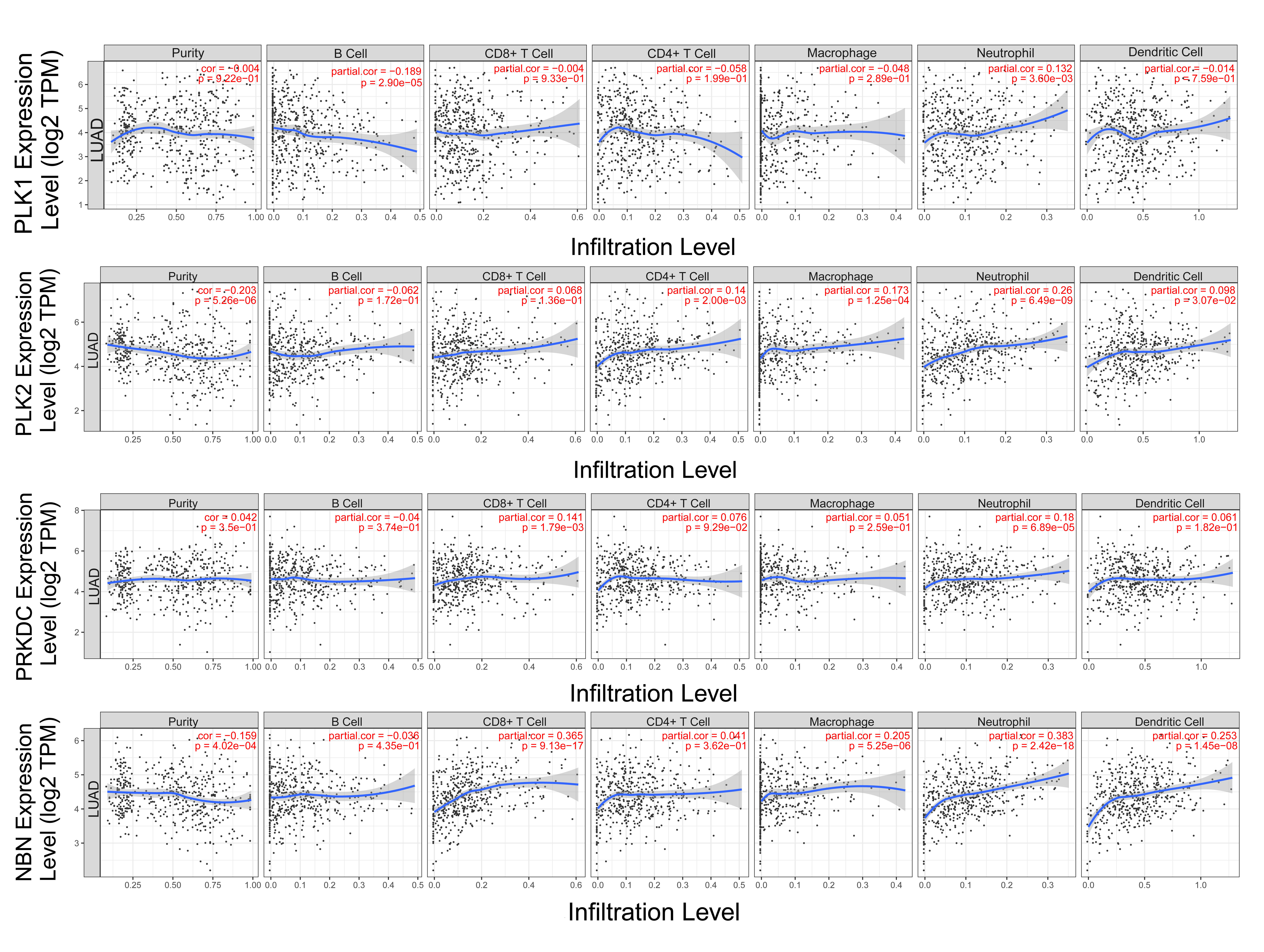


**Additional file 2: Fig. S3** The correlation analysis for 4 DCGs expression with infiltration levels of different immune cells in LUAD.





**Additional file 2: Fig. S4** Immunoactivity analysis of two risk groups in GEO cohorts. (**A**, **C**) The composition of 22 types of tumor-infiltrating immune cells in two risk groups. (**B**, **D**) The expression of 27 immune checkpoints in two risk groups.





**Additional file 2: Fig. S5** Comparison of chemoradiotherapy response in TCGA-LUAD. (**A**, **K**) The ssGSEA scores of 16 DDR pathways in the two risk groups. (**B**, **L**) The ssGSEA scores of X-ray and UV response in the two risk groups. (**C**-**F**, **M**-**P**) The sensitivity analysis of LUAD common chemotherapy agents (Cisplatin, Crizotinib, Erlotinib and Nilotinib) in two risk groups. (**G**-**J**, **Q**-**T**) The sensitivity analysis of other cancers common chemotherapy agents (Axitinib, Camptothecin, Etoposide and Gemcitabine).
